# A chronic whole cigarette smoke extract model reveals redox–mitochondrial adaptation in human lung epithelial and organoid models

**DOI:** 10.1038/s12276-026-01743-x

**Published:** 2026-06-08

**Authors:** Joo-Eun Lee, Dahye Lee, Jihyun Lee, Sung-Joon Han, Sung Hyun Kang, Ryeo-Eun Go, Jihyun Kwon, Younjhin Ahn, Mi Jung Lim, Mahn Jae Lee, Hee Min Yoo, Da Hyun Kang, Jeong Eun Lee, Dongil Park, Chaeuk Chung

**Affiliations:** 1 Division of Pulmonology and Critical Care Medicine, Department of Internal Medicine, College of Medicine, Chungnam National University, Daejeon, Republic of Korea; 2 Biomedical Convergence Research Institute, Chungnam National University Hospital, Daejeon, Republic of Korea; 3 Division of Thoracic and Cardiovascular Surgery, College of Medicine, Chungnam National University, Daejeon, Republic of Korea; 4 Division of Climate Change and Health Hazard, Department of Health Hazard Response, Korea Disease Control and Prevention Agency, Cheongju, Republic of Korea; 5 Genomics Department, Keyomics Co. Ltd., Yuseong-gu, Republic of Korea; 6 Biometrology Group, Korea Research Institute of Standards and Science (KRISS), Daejeon, Republic of Korea; 7 Department of Precision Measurement, University of Science and Technology (UST), Daejeon, Republic of Korea

**Keywords:** Mechanisms of disease, Experimental models of disease

## Abstract

Cigarette smoke (CS) imposes continuous oxidative and electrophilic stress that disrupts cellular homeostasis in the lung. While acute smoking exposure induces transient antioxidant responses, how the epithelial system adapts to chronic smoke remains poorly defined. Here we developed a chronic whole CS extract (WCSE) model that integrates both gaseous and particulate fractions to reproduce the complexity of long-term smoking exposure. Using bronchial epithelial cells and human lung organoids, we demonstrate that chronic WCSE exposure induces a coordinated redox–mitochondrial adaptation, supporting survival under persistent oxidative stress. Chronically exposed cells (T-B2B) exhibited reduced apoptosis, enhanced S-phase entry and fragmented but functionally preserved mitochondria characterized by a stable membrane potential and restrained reactive oxygen species accumulation. Whole-exome sequencing revealed oxidative mutational signatures in two-dimensional and organoid models, linking chronic oxidative adaptation with tobacco-associated genomic imprints. Mechanistically, NRF2 activity was sustained through post-translational stabilization and nuclear accumulation independent of KEAP1, accompanied by activated pAKT and suppressed pGSK3β activity. Human lung organoids recapitulated these adaptations, showing enlarged morphology, reduced apoptosis and nuclear NRF2 accumulation, consistent with a stress-tolerant, clonally persistent phenotype. Together, these findings establish a chronic WCSE platform that models early epithelial adaptation to CS and uncovers NRF2-dependent redox remodeling as a key mechanism of long-term survival in smoking-related pulmonary injury.

## Chronic cigarette smoke triggers lung epithelial adaptation

Cigarette smoke (CS) is a known carcinogen linked to various diseases, including cancer, due to its complex chemical composition. This study explores how chronic exposure to whole CS extract affects cellular and mitochondrial dynamics, focusing on the NRF2 antioxidant pathway. Researchers developed a model using human lung organoids and bronchial epithelial cells to simulate prolonged CS exposure, incorporating both particulate and gaseous components. They found that chronic whole CS extract exposure enhances cell survival, induces mitochondrial fragmentation and maintains mitochondrial membrane potential, suggesting a stress-resistant phenotype. Notably, NRF2 activity increased through protein stabilization and nuclear localization, promoting antioxidant defenses. These findings highlight the adaptive mechanisms cells employ under chronic oxidative stress, potentially contributing to premalignant changes. The study suggests that future research could explore therapeutic strategies targeting these adaptive pathways to mitigate smoking-related cancer risks.

This summary was initially drafted using artificial intelligence, then revised and fact-checked by the author.

## Introduction

Cigarette smoke (CS) is a well-established carcinogenic agent that affects not only respiratory diseases but also multiple organ system disorders^1^. It contains more than 4,000 identified chemical ingredients, including nicotine, ammonia, nitrogen oxides, hydrogen cyanide and trace metals. These compounds act synergistically to induce oxidative stress, DNA damage, chronic inflammation and cellular senescence, leading to malignant transformation^2–4^. Recent studies have demonstrated that long-term exposure to CS can promote abnormal cell proliferation, disrupt DNA repair systems and alter the tumor microenvironment, leading to enhanced cancer progression^4–6^. Moreover, CS has been reported to promote colorectal cancer progression by modulating gut microbial metabolites^7^. In lung cancer, CS has been shown to promote cell cycle progression and contribute to the aggressiveness of smoking-related non-small cell lung cancer^8^.

Among the components of CS, reactive oxygen species (ROS) play a crucial role in its pathogenic effects. Prolonged CS exposure leads to sustained ROS accumulation, overwhelming endogenous antioxidant systems and inducing chronic oxidative stress. Mitochondria, as the primary site of cellular respiration, are both a major source and a primary target of this ROS-induced damage. Consequently, alterations in mitochondrial dynamics and membrane potential are increasingly recognized as a key feature of cancer, contributing to metabolic reprogramming, resistance to apoptosis and enhanced cell survival^9^.

Smoking also leaves a genomic footprint. Large-scale sequencing has connected tobacco exposure with a single-base substitution (SBS) signature indicative of oxidative injury and DNA-repair imbalance^10^. To counteract oxidative damage, cells activate the transcription factor NRF2 (nuclear factor erythroid related factor 2), which regulates antioxidant and detoxifying genes, such as *NQO1* and *HO-1*^1^. Under basal conditions, NRF2 is rapidly degraded; however, when subject to oxidative stress, the protein is stabilized and promoted to accumulate in the nucleus, maintaining antioxidant defense under chronic stress^11^.

While transient activation of NRF2 during acute oxidative stress is well established, how NRF2 activity is maintained under chronic cigarette exposure remains poorly understood. Previous experimental models investigating CS-induced cellular responses have primarily relied on either particulate-phase cigarette condensate or gas-phase CS extract, typically applied in short-term exposure settings. However, these models capture only a limited fraction of the whole CS and therefore fail to represent the integrated chemical complexity and sustained oxidative burden associated with chronic smoking^12,13^. Furthermore, most studies have been conducted in two-dimensional culture systems or animal models, which cannot fully recapitulate the structural and cellular complexity of the human airway epithelium. As a result, these approaches predominantly model acute cytotoxic or inflammatory responses rather than the long-term adaptive remodeling induced by chronic smoke exposure. Although three-dimensional lung organoid systems have recently emerged as promising platforms for studying human airway biology^14^, their application to sustained CS exposure remains limited.

To overcome these limitations, we developed a whole CS extract (WCSE) model that combines both particulate and gaseous components to more accurately represent the physiological complexity of a chronic exposure platform. Furthermore, we established a human lung organoid-based chronic exposure platform, enabling the investigation of cigarette-induced adaptation in a biologically relevant, three-dimensional platform.

Collectively, this study establishes a chronic WCSE exposure platform that integrates both particulate and gaseous components of CS in human airway epithelial and lung organoid systems. Using this chronic WCSE exposure model system, we demonstrated that chronic WCSE exposure promotes cellular survival and induces mitochondrial fragmentation while preserving membrane potential, and that it generates mutational signatures characteristic of oxidative stress. We further reveal that NRF2 activity is maintained post-translationally through protein stabilization and nuclear localization, resulting in persistent antioxidant activation. Together, these adaptations converge to produce a stress-tolerant, clonally expanding phenotype that resists apoptosis and maintains persistent antioxidant activation under chronic cigarette exposure.

## Materials and methods

### Preparation of the WCSE

The WCSE was prepared following the standardized WCSE methods described by a previous report^15^ with minor modifications. In summary, 3R4F reference cigarettes were conditioned for 48 h (22 °C, 60% relative humidity) and smoked using an SG-300 smoking machine under the Health Canada Intense regimen (55 ml puff volume, 2 s duration, 30 s interval, 100% vent blocking). Mainstream smoke was passed through a 44-mm Cambridge filter to collect total particulate matter (TPM) and subsequently bubbled into Dulbecco’s phosphate-buffered saline to capture the gas-phase fraction. TPM-loaded filters were extracted in 5 ml of methanol, and the solvent was removed by nitrogen evaporation; the resulting residue was dissolved in 1% dimethyl sulfoxide and combined with the Dulbecco’s phosphate-buffered saline fraction to generate WCSE containing both particulate and gaseous components. The prepared WCSE was aliquoted and stored at −80 °C, ensuring component stability as validated in the referenced method.

### Cell culture and chronic WCSE exposure

BEAS-2B and A549 cells were maintained in Dulbecco’s modified Eagle medium or RPMI-1640 with 10% fetal bovine serum and antibiotics at 37 °C and 5% CO₂. Chronically exposed BEAS-2B cells (T-B2B) were generated through continuous treatment with 100 µg/ml WCSE for 40 weeks.

### Human lung organoid culture and long-term exposure

Normal lung tissues were dissociated, filtered through a 70-µm cell strainer, subjected to red blood cell lysis and embedded in basement membrane extract. Organoids were seeded as 50-µl domes (~10,000 cells per dome) and maintained in lung organoid medium (Supplementary Table 1). The medium was changed every 3 days. The lung organoid culture system was established following previously described human lung organoid protocols with minor modifications^16,17^. Chronically exposed organoids (T-hLO) were established through long-term WCSE treatment. Organoid experiments were performed using three independent donor-derived organoid lines, and representative lines (hLO-08, hLO-12 and hLO-26) are shown in Supplementary Fig. 4a–c and Supplementary Table 2. A schematic overview of the organoid establishment workflow is provided in Supplementary Fig. 3a.

### Histology and immunofluorescence

Organoids were washed to remove basement membrane extract, fixed in 4% paraformaldehyde, embedded in Histogel, paraffin-processed, sectioned (4 µm) and stained according to standard hematoxylin and eosin (H&E) protocols. For immunofluorescence, organoids or cultured cells were fixed, permeabilized with 0.1% Triton X-100, blocked with 3% bovine serum albumin and incubated with primary antibodies (for example, NRF2) overnight at 4 °C. Alexa Fluor-conjugated secondary antibodies were used, and images were acquired using a confocal microscope.

### Viability, apoptosis and cell-cycle analysis

Cell viability following WCSE exposure was quantified using Cell Counting Kit-8 (CCK-8) and 3D-Titer Glo viability assay. Apoptosis in 2D cells and organoids was assessed by Annexin V/7-AAD flow cytometry. Cell-cycle distribution was analyzed through Hoechst 33342 and Ki-67 staining.

### WES of B2B and T-B2B cells

Whole-exome sequencing (WES) was performed for BEAS-2B and chronically exposed T-B2B cells using an Illumina paired-end platform (Macrogen). Preprocessed BQSR BAM files were aligned to the GRCh38 reference genome and analyzed following the GATK Best Practices workflow. Somatic variants were identified using Mutect2 with paired B2B–T-B2B comparison, followed by FilterMutectCalls. Common germline variants (allele frequency ≥1%) were filtered using dbSNP138 and af-only gnomAD (hg38). Variant annotation was performed using VEP (v114) and Funcotator (hg38).

### WGS of hLO and T-hLO organoids

Whole-genome sequencing (WGS) of hLO and T-hLO was performed using Illumina NovaSeq X (151-bp paired-end). Approximately 120 GB of raw data were generated per sample (>60× depth). Reads were aligned to GRCh38 and processed using GATK Best Practices for WGS, including duplicate marking, base recalibration, and variant calling via Mutect2 with matched normal-exposed pairs. Germline variant filtering and functional annotation were performed using the same dbSNP138, gnomAD, VEP and Funcotator pipelines used for WES.

### TCGA analysis

The Cancer Genome Atlas (TCGA) lung adenocarcinoma (LUAD) and lung squamous cell carcinoma (LUSC) datasets were obtained from the GDC using TCGAbiolinks. HTSeq–Fragments Per Kilobase of transcript per Million mapped reads (FPKM) gene expression matrices were log₂-transformed, and tumor-normal differential expression analyses were performed in R. Key oxidative-stress-related and NRF2-pathway genes were evaluated across both TCGA cohorts.

### Mitochondrial morphology and function

Mitochondrial morphology was assessed by MitoTracker Red staining, and mitochondrial area, length and circularity were quantified from fluorescence images. Mitochondrial membrane potential was measured using tetramethylrhodamine methyl ester (TMRM) by flow cytometry. Total cellular ROS and mitochondrial ROS were quantified using DCFDA and MitoSOX, respectively.

### Western blotting

Cells were lysed in a protein lysis buffer, and protein concentrations were determined by Bradford assays. Equal amounts of protein (30 µg) were resolved by sodium dodecyl sulfate–polyacrylamide gel electrophoresis, transferred to polyvinylidene fluoride membranes, blocked in 5% skim milk and probed with antibodies against β-actin, NRF2, KEAP1, HO-1, AKT, p-AKT, GSK3β, p-GSK3β and NQO1. Horseradish peroxidase-conjugated secondary antibodies and enhanced chemiluminescence reagents were used for detection.

### Nuclear–cytoplasmic fractionation

Nuclear and cytoplasmic fractions were isolated using the NE-PER extraction kit. Fraction purity was confirmed by western blotting with Lamin A/C (nuclear marker) and β-actin (cytoplasmic marker).

### siRNA-mediated knockdown

T-B2B cells were transfected with NRF2-targeting short interfering RNAs (siRNAs) (20 nM) or a nontargeting control using RNAiMAX. After transfection, cells were exposed to WCSE (400 µg/ml) for 24 h, and apoptosis was quantified by morphological analysis and flow cytometry.

### Quantitative RT–PCR

Total RNA was extracted using TRIzol, and cDNA was synthesized using oligo(dT) primers. Gene expression of NRF2, KEAP1, NQO1 and HO-1 was quantified by quantitative PCR using gene-specific primers.

### Organoid size analysis

Organoid size and number were quantified using the GelCount system, which automatically detects and measures organoids from bright-field images acquired from 96-well plates.

### Statistical analysis

All experiments were performed in triplicate. Data are presented as the mean ± standard error of the mean (s.e.m.). Statistical significance between two groups was determined using a *t*-test.

## Results

### Long-term WCSE exposure increases cell survival and promotes S-phase progression in bronchial epithelial cells

**Fig. 1:**
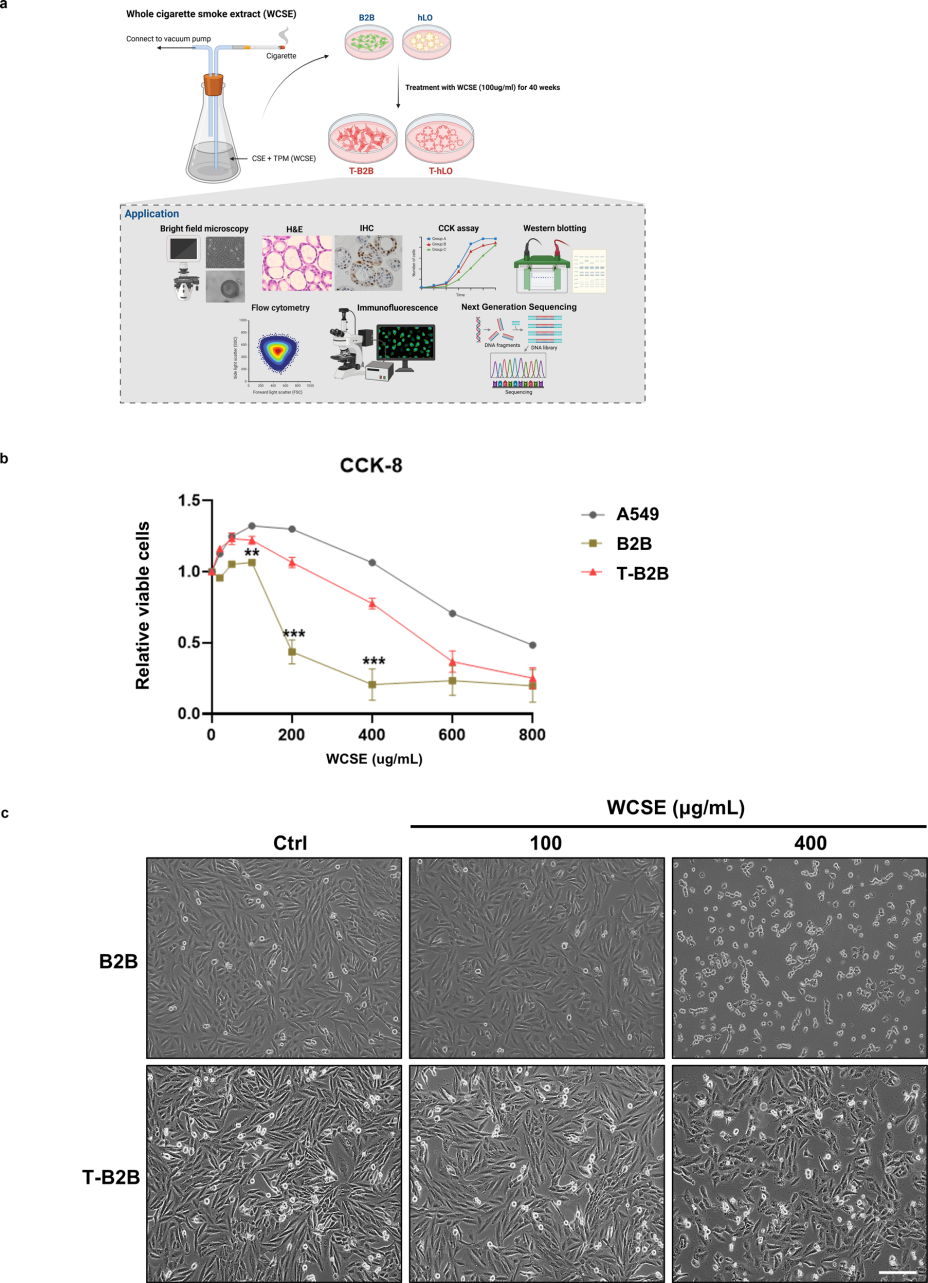

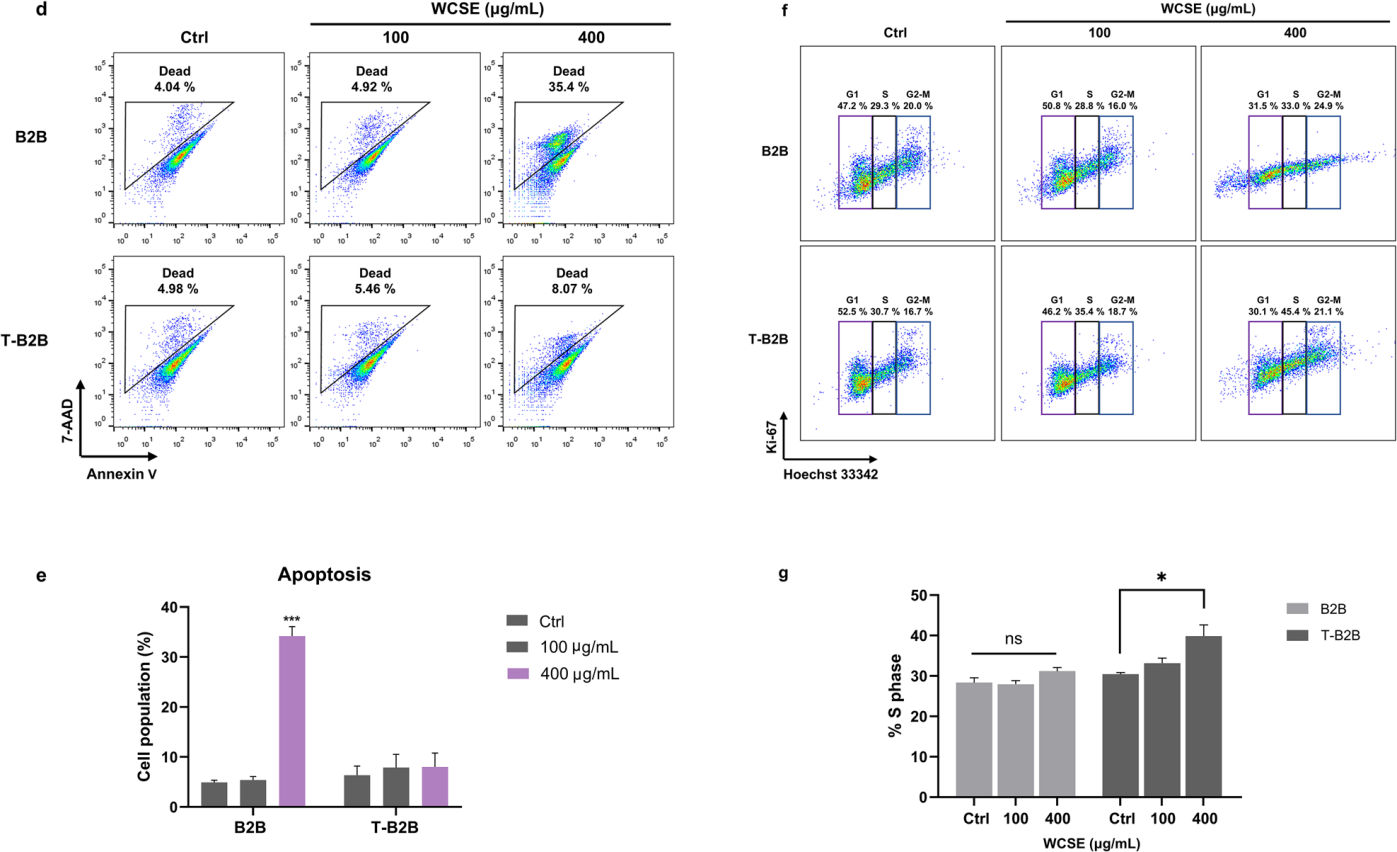
Long-term WCSE exposure enhances survival and suppresses apoptosis. **a** Schematic overview of the chronic cigarette exposure model and experimental applications. **b** A549, B2B and T-B2B cells were treated with increasing concentrations of WCSE (0–800 μg/ml) for 24 h, measured using the CCK-8 assay. T-B2B maintained significantly higher viability than B2B. **c** Representative phase-contrast images of B2B and T-B2B after 24 h WCSE exposure (Ctrl, 100, 400 μg/ml). T-B2B retained normal adherent morphology, whereas B2B exhibited cell shrinkage and detachment. Scale bar, 100 μm. **d** Representative flow cytometry plots of Annexin V/7-AAD staining in B2B and T-B2B cells after WCSE treatment (Ctrl, 100, 400 μg/ml). **e** Quantification of apoptotic populations. **f** Cell-cycle analysis was determined by Hoechst 33342/ Ki-67 staining. **g** Quantification of cell-cycle distribution.T-B2B increased S-phase and reduced G0/G1 populations compared with B2B. Data are presented as mean ± s.e.m. (*n* = 3). Statistical significance was analyzed by a *t*-test.

A schematic overview of the experimental design is shown in Fig. 1a. WCSE was prepared by combining both gas-phase (CSE) and particulate-phase (TPM) fractions collected from mainstream CS, providing a physiologically relevant whole-smoke extract for chronic exposure experiments (Supplementary Fig. 1a). To determine whether long-term WCSE exposure contributes to premalignant adaptation, we generated a chronically exposed bronchial epithelial cell line (T-B2B) by continuously treating BEAS-2B (B2B) cells with 100 μg/ml WCSE over 40 weeks. To assess the effect of long-term WCSE exposure on cell viability, B2B, T-B2B and A549 cells were treated with increasing concentrations of WCSE. The CCK-8 assay revealed a dose-dependent decrease in viability in B2B cells; however, T-B2B and A549 cells maintained significantly higher survival rates (Fig. 1b). In addition, B2B cells showed morphological changes such as shrinkage and detachment, while T-B2B cells retained an adherent and elongated morphology (Fig. 1c). When examining apoptosis, we found that apoptotic cell populations in B2B samples increased when treated with WCSE, but not in T-B2B samples. These results demonstrate that chronic exposure confers resistance to apoptosis (Fig. 1d, e). As enhanced cell survival might be due to accelerated proliferation, we analyzed the cell cycle distribution. In T-B2B samples, there was a higher S-phase population and a decrease in G0/G1 phases compared with B2B (Fig. 1f, g). Together, these results indicate that long-term WCSE exposure enhances stress tolerance, suppresses apoptosis and promotes sustained proliferation.

### Long-term WCSE exposure induces mitochondrial fragmentation and functional remodeling

**Fig. 2:**
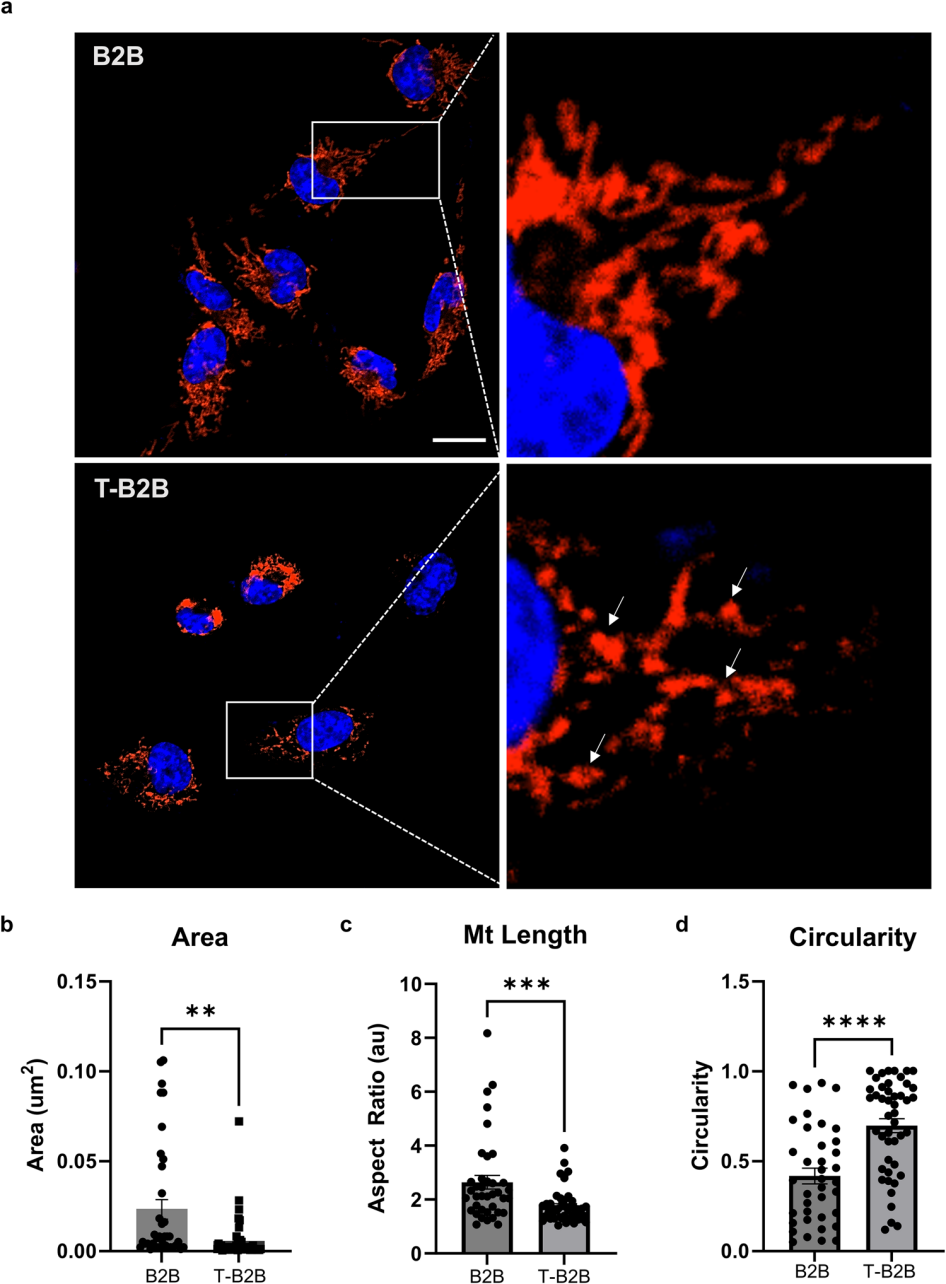

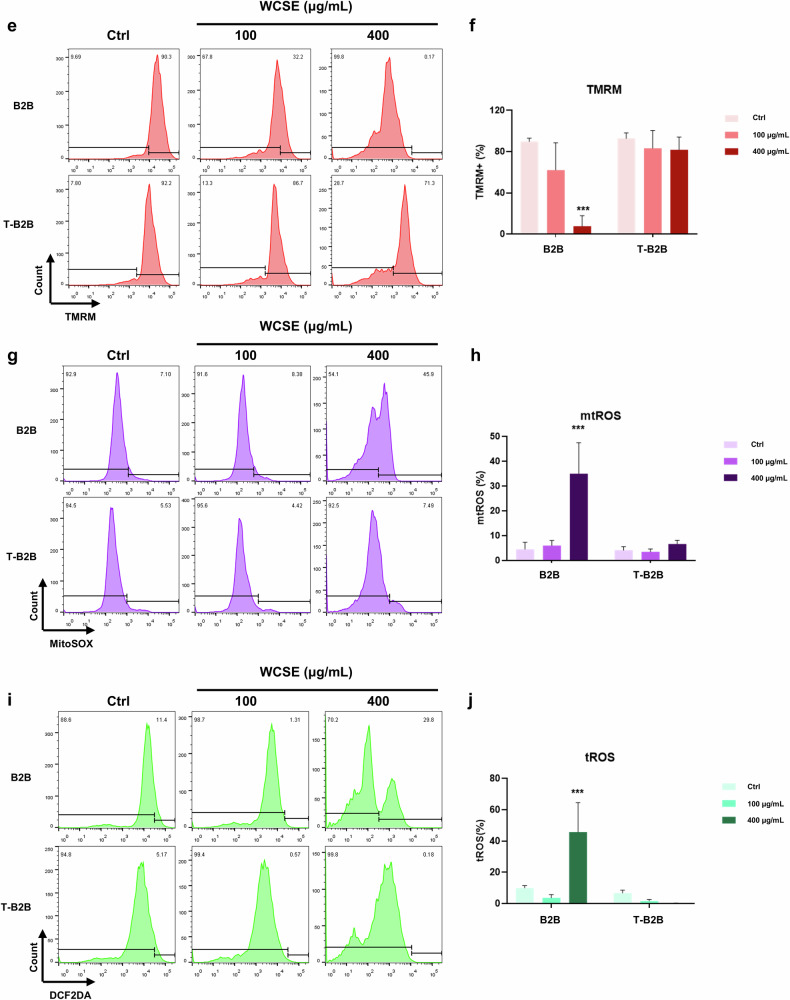
Long-term WCSE exposure induced mitochondrial fragmentation and functional adaptations. **a** Representative MitoTracker Red staining of B2B and T-B2B cells showing mitochondrial morphology. Enlarged images highlight fragmented mitochondria in T-B2B (indicated by white arrows). Scale bar, 100 μm. **b** Quantitative analysis of mitochondrial area. **c** Quantitative analysis of mitochondrial length. **d** Quantitative analysis of mitochondrial circularity. T-B2B displayed reduced area and length with increased circularity. **e** Representative flow-cytometric plots of mitochondrial membrane potential using TMRM. **f** Quantification of mitochondrial membrane potential. Acute WCSE exposure reduced potential in B2B but not in T-B2B. **g** Representative flow-cytometric plots of mitochondrial ROS measured using MitoSOX. **h** Quantification of mitochondrial ROS levels. Mitochondrial ROS increased in B2B under WCSE but remained low in T-B2B. **i** Representative flow-cytometric plots of total ROS measured using DCF-2DA. **j** Quantification of total ROS levels. T-B2B exhibited lower ROS accumulation compared with B2B. Data are presented as mean ± s.e.m. (*n* = 3). Statistical significance was analyzed by a *t*-test.

Mitochondrial dynamics play a critical role in cancer progression, and mitochondrial fragmentation has been associated with enhanced stemness and invasiveness in malignant cells^9^. Based on these reports, we conducted an investigation and found that long-term WCSE exposure alters mitochondrial morphology and function in the T-B2B model. MitoTracker staining revealed morphological differences between B2B and T-B2B cells. In B2B, mitochondria formed an elongated and interconnected network. By contrast, in T-B2B, they appeared shorter and more fragmented (Fig. 2a). Quantitative analysis confirmed a substantial decrease in mitochondrial area and length, along with an increase in circularity in T-B2B compared with B2B (Fig. 2b–d), indicating enhanced mitochondrial fragmentation. To assess functional effects, mitochondrial membrane potential as well as mitochondrial and total ROS were analyzed following WCSE treatment. In B2B, acute WCSE exposure markedly decreased membrane potential (Fig. 2e, f) and increased ROS production (Fig. 2g–j), while T-B2B maintained mitochondrial membrane potential and exhibited a decrease in ROS accumulation (Fig. 2e–j). These findings suggest that chronic WCSE exposure reprograms mitochondrial dynamics and function, leading to a mitochondria-dependent stress-resistant adaptive state.

### Long-term WCSE exposure induces a distinct mutational landscape characterized by oxidative stress

**Fig. 3:**
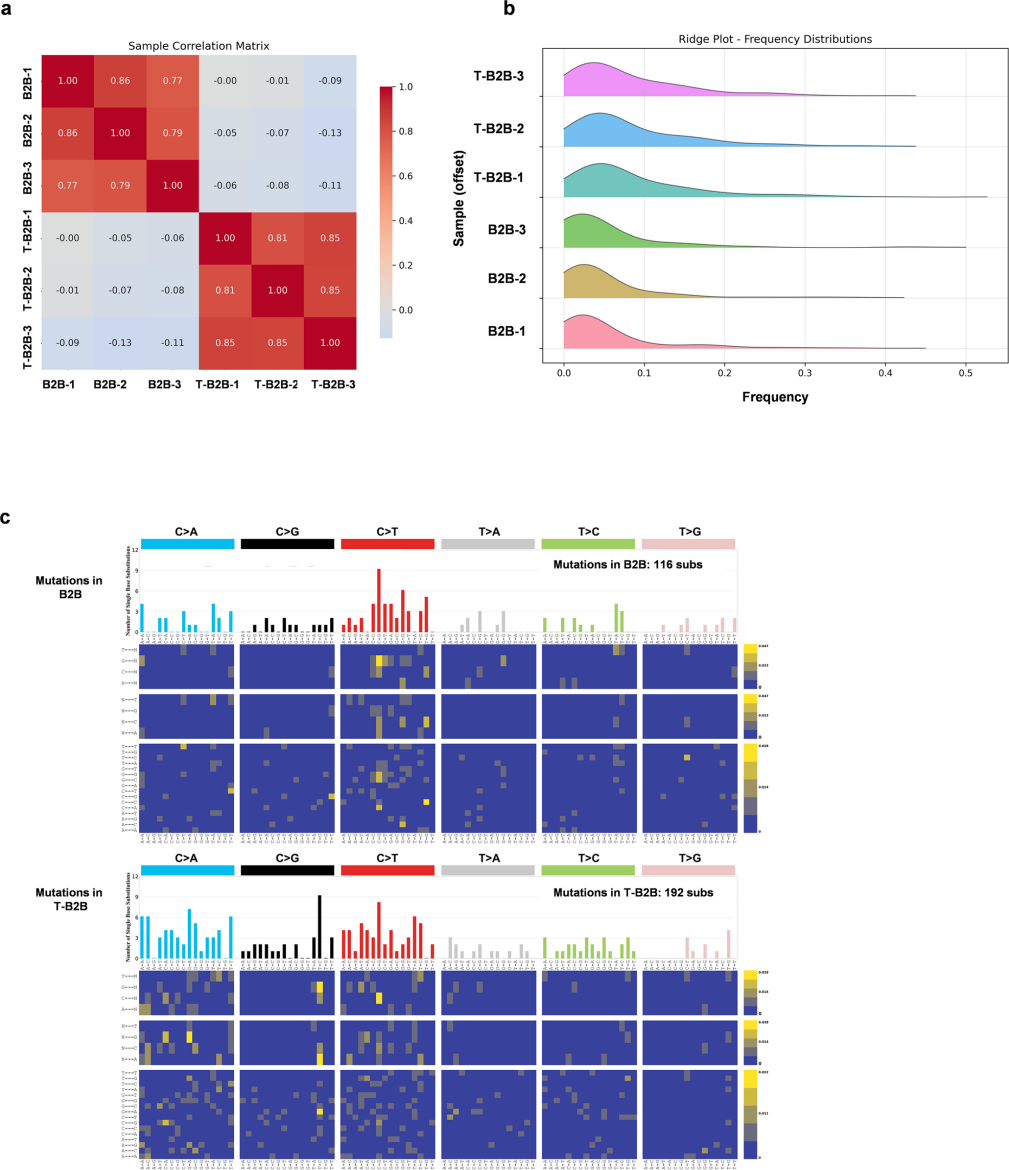

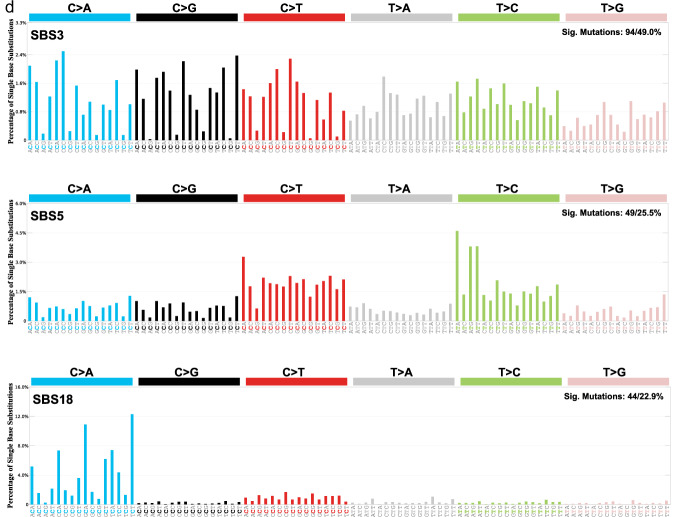
WES identifies oxidative stress-associated mutational signatures following long-term WCSE exposure. **a** Correlation matrix of WES data showing distinct clustering of B2B and T-B2B cells after chronic WCSE exposure. **b** Ridge plots of variant allele frequency distributions in B2B and T-B2B cells. T-B2B exhibited broader and higher-frequency variants compared with B2B. **c** Base substitution patterns of six classes (C > A, C > G, C > T, T > A, T > C, T > G). T-B2B showed an increase in C > A and C > T substitutions. **d** Mutational signature analysis showing enrichment of SBS3, SBS5, and SBS18 in T-B2B cells. SBS18 corresponds to oxidative DNA damage, SBS5 represents long-term mutational processes, and SBS3 is related to defective double-strand break repair.

To investigate the genomic alterations caused by chronic CS exposure, we performed WES of B2B and T-B2B cells. Correlation analysis revealed a clear genomic separation between the two groups, indicating divergence after prolonged WCSE exposure (Fig. 3a). Ridge distribution analysis further showed a broader and higher variant frequency spectrum in T-B2B, consistent with an increased mutation load (Fig. 3b). Analysis of SBSs demonstrated that T-B2B accumulated higher mutational burden, with 192 substitutions compared with 116 in the parental B2B cells (Fig. 3c). Notably, the mutation profile of T-B2B cells was increased in C > A transversions, a class of mutation strongly associated with oxidative DNA damage. This finding is consistent with the known ability of CS to induce a state of chronic oxidative stress through the production of ROS^18^. To better understand the underlying mutational processes, we identified three predominant signatures, SBS3, SBS5 and SBS18, in T-B2B (Fig. 3d). These signatures have been reported in smoking-related lung cancer. They are associated with oxidative DNA damage and defective DNA repair. This finding is consistent with large-scale genomic data from patients with non-small cell lung cancer, where SBS18 and SBS5 were significantly enriched in smoking-high clusters compared with smoking-low clusters, reflecting the mutational impact of chronic cigarette exposure^10^. Together, these results support a mechanistic connection between CS-induced oxidative stress and the genomic alterations that contribute to premalignant progression.

### TCGA analysis reveals NRF2 activation and antioxidant gene upregulation in lung squamous carcinoma and long-term WCSE-exposed cells

**Fig. 4:**
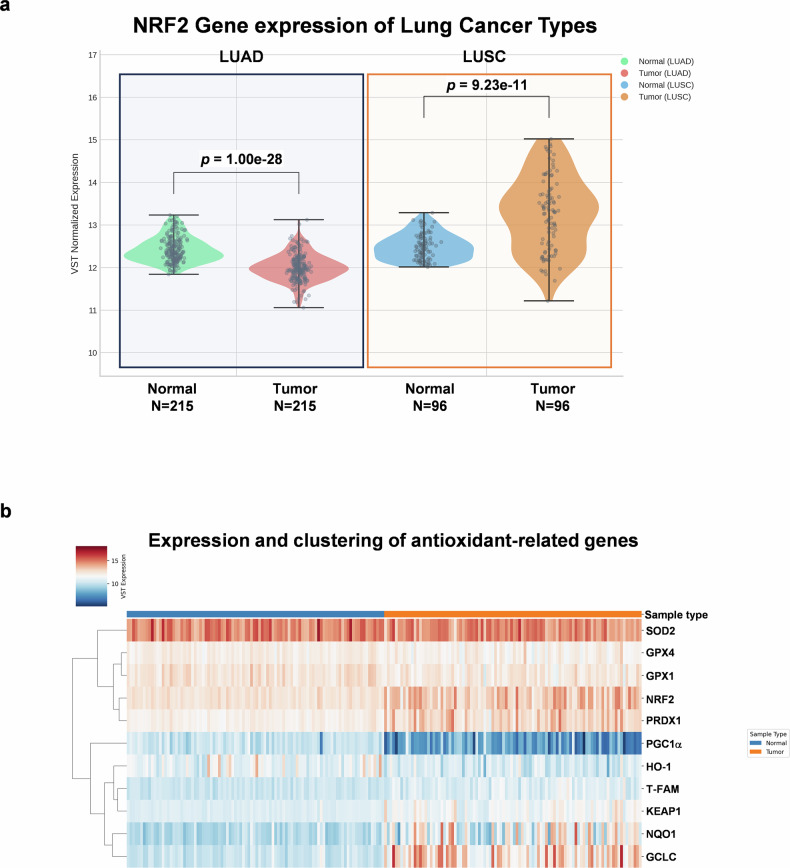

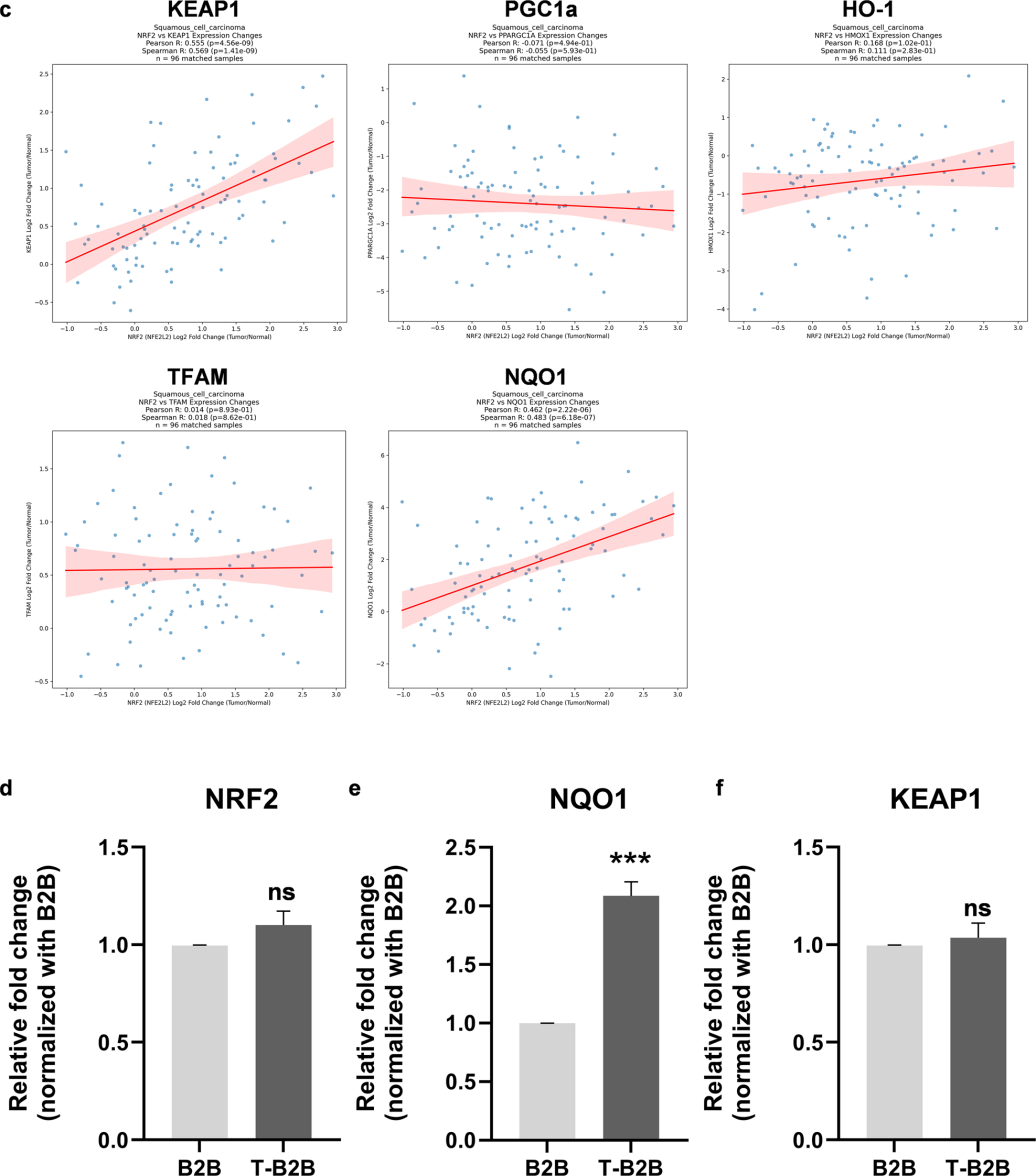
TCGA analysis and experimental validation reveal activation of the NRF2-antioxidant defense pathway following chronic cigarette exposure. **a** Expression of *NRF2* in LUAD and LUSC from the TCGA dataset*. NRF2* expression was significantly increased in LUSC and decreased in LUAD compared with normal tissues. **b** Heatmap showing coordinated upregulation of antioxidant-related genes (*NQO1*, *GCLC*, *HMOX1*, *GPX1* and *GPX4*) in LUSC compared with normal tissues. **c** Correlation analysis demonstrating positive associations among *NQO1*, *PGC1a* and *HO-1* expression in LUSC samples. **d** Quantitative RT–PCR analysis of *NRF2* expression in B2B and long-term WCSE-exposed T-B2B cells. *NRF2* expression remained unchanged. **e** Quantitative RT–PCR analysis of *NQO1* expression in B2B and T-B2B cells. *NQO1* expression was significantly elevated in T-B2B. **f** Quantitative RT–PCR analysis of *KEAP1* expression in B2B and T-B2B cells. *KEAP1* expression remained unchanged. Data are presented as mean ± s.e.m. (*n* = 3). Statistical significance was analyzed by a *t*-test.

Chronic cigarette exposure induced mitochondrial alteration and oxidative stress-associated mutational signatures. We examined the expression of NRF2^11^, a master regulator of the cellular antioxidant response. To assess the clinical relevance of this pathway, we analyzed gene expression profiles from the TCGA lung cancer cohort. In LUSC, a subtype strongly linked to smoking, *NRF2* was significantly upregulated (*P* < 0.001). By contrast, *NRF2* expression was downregulated in LUAD (*P* < 0.001) compared with normal tissues (Fig. 4a). Based on the pronounced upregulation of *NRF2* in LUSC, we focused our subsequent analysis on this subtype. In line with the increase in *NRF2*, we observed a distinct clustering and co-upregulation of multiple NRF2-target antioxidant genes, including *NQO1*, *GCLC*, *HO-1 (HMOX1)* and the *GPX* family, in LUSC compared with normal tissues (Fig. 4b). To further explore the relationship within this pathway, we determined a positive correlation between the mRNA expression of *NRF2* and its target genes, including *NQO1*, *PGC1* and *HO-1* in the LUSC cohort (Fig. 4c), suggesting that CS activates the antioxidant defense network. Next, to validate these transcriptomic patterns, we compared NRF2 pathway gene expression in B2B and T-B2B cells. *NQO1* levels were dramatically elevated in T-B2B, while *NRF2* and *KEAP1* expression remained unchanged (Fig. 4d-f). Although *NRF2* expression was unchanged, the induction of *NQO1* reflects functional activation of the NRF2-antioxidant axis as an adaptive response to chronic cigarette exposure. Consistent with these findings, analysis of additional antioxidant genes revealed that expression of *PGC1α*, a key regulator of mitochondrial biogenesis, was significantly increased, while *GPX1* and *GPX4* remained unchanged (Supplementary Fig. 2a–c). Together, these results indicate that chronic cigarette exposure enhances antioxidant defense through *NQO1* induction and *PGC1α*-mediated mitochondrial adaptation.

### Chronic cigarette exposure stabilizes the NRF2 protein and promotes its nuclear accumulation

**Fig. 5:**
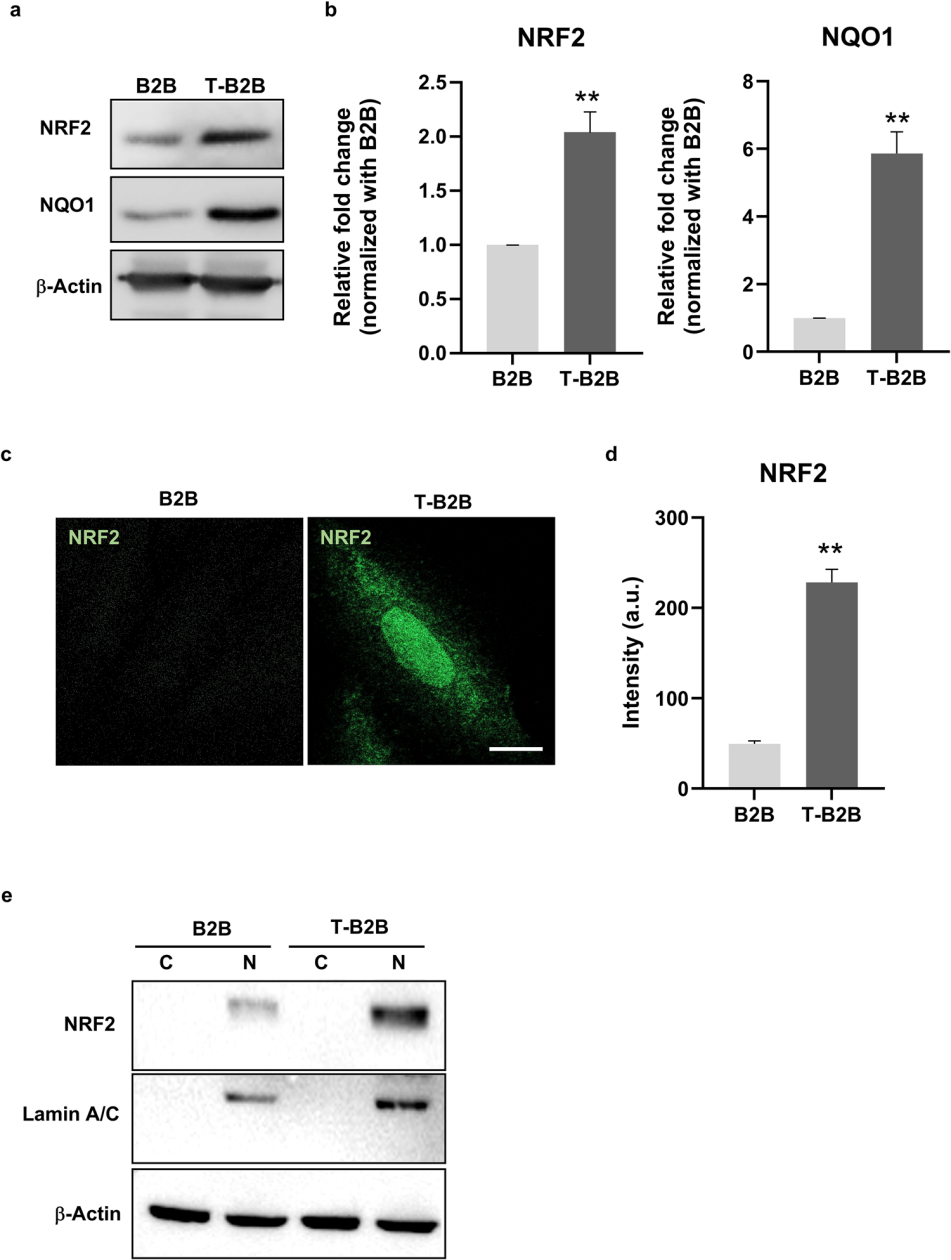
Long-term WCSE exposure enhances NRF2 protein stability and nuclear translocation in T-B2B cells. **a** Immunoblot analysis of NRF2 and its downstream target NQO1 in B2B and T-B2B cells shows increased levels of both proteins in T-B2B. **b** Quantification of band intensity indicates upregulation of NRF2 and NQO1 in T-B2B cells. **c** Immunofluorescence images showing increased NRF2 signal intensity and nuclear localization in T-B2B cells. Scale bar, 20 μm. **d** Quantification of NRF2 fluorescence confirms a significant elevation in T-B2B cells. **e** Subcellular fractionation and immunoblotting reveal NRF2 enrichment in the nuclear (N) fraction of T-B2B cells, with Lamin A/C and β-Actin representing nuclear and cytoplasmic markers. Data are presented as mean ± s.e.m. (*n* = 3). Statistical significance was analyzed by a *t*-test.

Based on our finding that *NRF2* target genes were induced without a corresponding increase in *NRF2* mRNA, we hypothesized that NRF2 activity was regulated at the post-translational level. To determine whether NRF2 was indeed regulated at the protein level, we examined the expression and subcellular localization of the NRF2 protein. Western blot analysis revealed that the total protein levels of both NRF2 and its target, NQO1, were significantly increased in T-B2B cells compared with control B2B cells (Fig. 5a, b). Next, we investigated the cellular localization of NRF2 as a nuclear translocation is essential for its function as a transcription factor. Immunofluorescence staining showed a dramatic accumulation of NRF2 within the nucleus of T-B2B cells, whereas the signal in B2B cells was negligible (Fig. 5c, d). Consistently, subcellular fractionation confirmed a robust presence of NRF2 in the nuclear fraction of T-B2B cells, whereas it was reduced in the B2B cells (Fig. 5e). Taken together, these data demonstrate that chronic cigarette exposure leads to the stabilization and subsequent nuclear translocation of the NRF2 protein, resulting in the functional activation of its antioxidant pathway.

### Chronic cigarette exposure promotes clonal expansion and mitochondrial adaptation in lung organoids

**Fig. 6:**
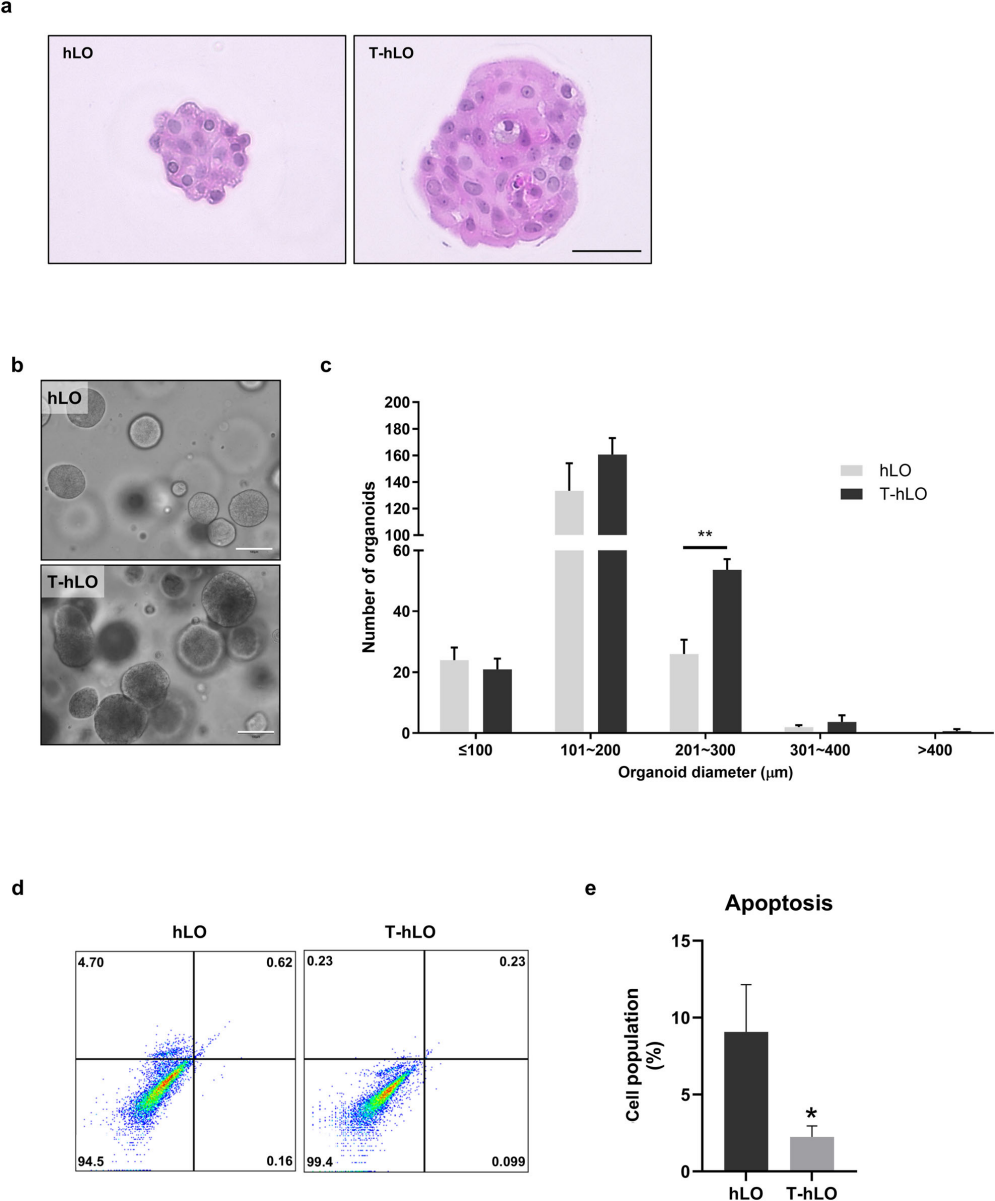

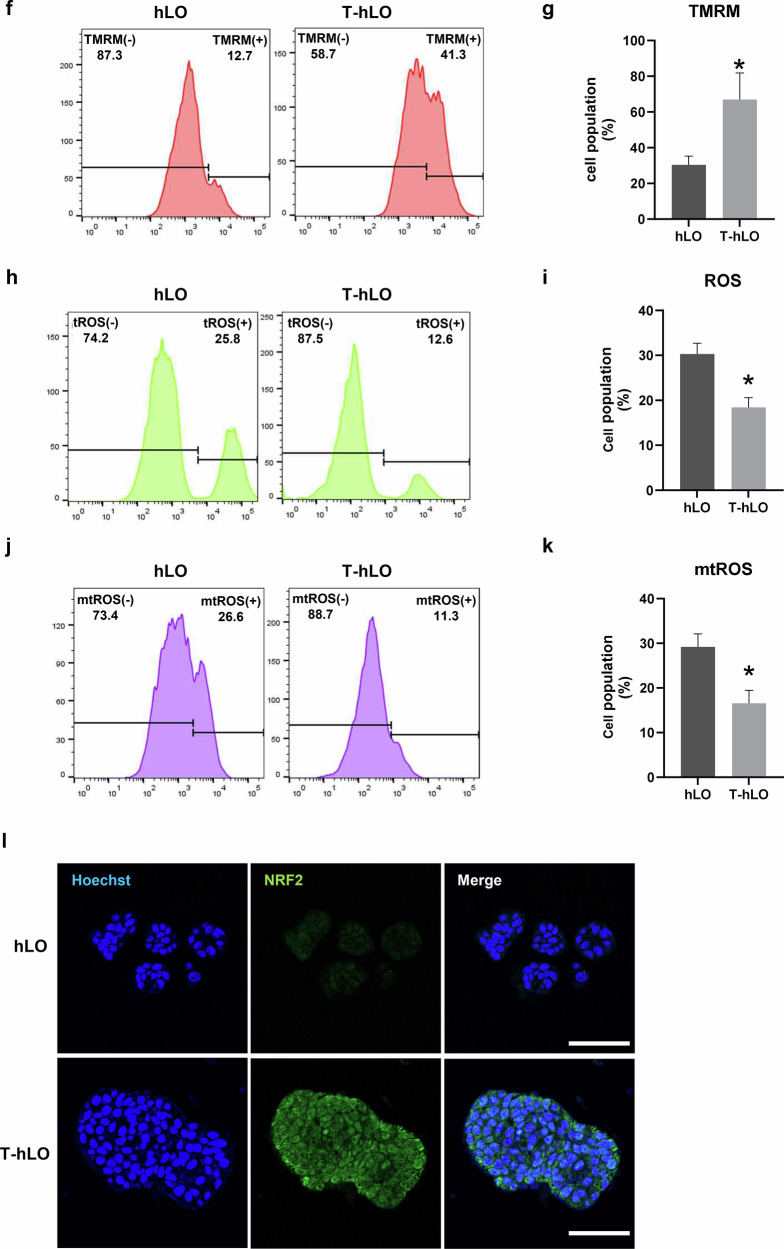
Chronic cigarette exposure promotes clonal expansion and antioxidant adaptation in normal lung organoids. **a** Representative H&E-stained images showing morphological differences between control (hLO) and chronically exposed (T-hLO) organoids. Scale bar, 100 μm. **b** The morphology of normal human lung organoids (hLOs) and long-term WCSE-exposed organoids (T-hLOs). Scale bar, 100 μm. **c** Quantification of organoid size. **d** Representative flow-cytometry plots of ANNEXIN V/7-AAD staining in hLOs and T-hLOs. **e** Quantification of apoptotic populations. T-hLOs exhibited a significantly lower apoptotic population compared with hLOs. **f** Representative flow-cytometry plots of mitochondrial membrane potential after 100 μg/ml WCSE treatment. **g** Quantification of mitochondrial membrane potential. T-hLOs showed higher mitochondrial membrane potential. **h** Representative flow-cytometry plots of total ROS measured using DCF-2DA. **i** Quantification of total ROS levels. **j** Representative flow-cytometry plots of mitochondrial ROS measured using MitoSOX. **k** Quantification of mitochondrial ROS levels. **l** Representative immunofluorescence images showing NRF2 (green) localization in control (hLO) and long-term WCSE-exposed organoids (T-hLO). Data are presented as mean ± s.e.m. (*n* = 3 independent donor-derived organoid lines). Statistical significance was analyzed by a *t*-test. Scale bar, 100 μm.

To validate our findings in a more physiologically relevant model, we established a long-term WCSE-exposed normal human lung organoid (T-hLO) model. Based on viability analyses, 100 μg/ml WCSE was selected as it is a sublethal concentration that permits sustained exposure while maintaining organoid viability (Supplementary Fig. 5a). After chronic exposure, the morphology of T-hLOs was larger and more structurally robust than control hLOs (Fig. 6a, b). To quantify these morphological changes, we analyzed the size distribution of the organoids across both models after WCSE treatment. T-hLOs showed a markedly higher proportion of large organoids (>200 μm in diameter), indicating enhanced growth potential under chronic exposure (Fig. 6c). Also, we analyzed apoptosis and found that T-hLOs exhibited a significantly lower apoptotic cell population compared with hLOs (Fig. 6d, e). This demonstrates that chronic smoke exposure confers a survival advantage, leading to the selection and expansion of cells that have acquired resistance to apoptosis, which is a key hallmark of cancer^19^. Next, we assessed mitochondrial function after treatment with 100 μg/ml WCSE. T-hLOs maintained higher mitochondrial membrane potential (Fig. 6f, g) and lower levels of total and mitochondrial ROS (Fig. 6h–k) compared with hLO. Indeed, immunofluorescence analysis showed a substantial nuclear accumulation of NRF2 in T-hLOs (Fig. 6l). Genomic analysis of long-term WCSE-exposed organoids also revealed the presence of the tobacco-associated mutational signature SBS4 (Supplementary Fig. 7a, b). Overall, these findings demonstrate that chronic smoke exposure promotes the development of a stress-resistant adaptive state that mimics early features of carcinogenesis, characterized by acquired resistance to apoptosis and continuous activation of the NRF2 survival pathway.

### NRF2-dependent survival in T-B2B cells is mediated by uncoupling stress from the AKT–GSK3β axis

**Fig. 7:**
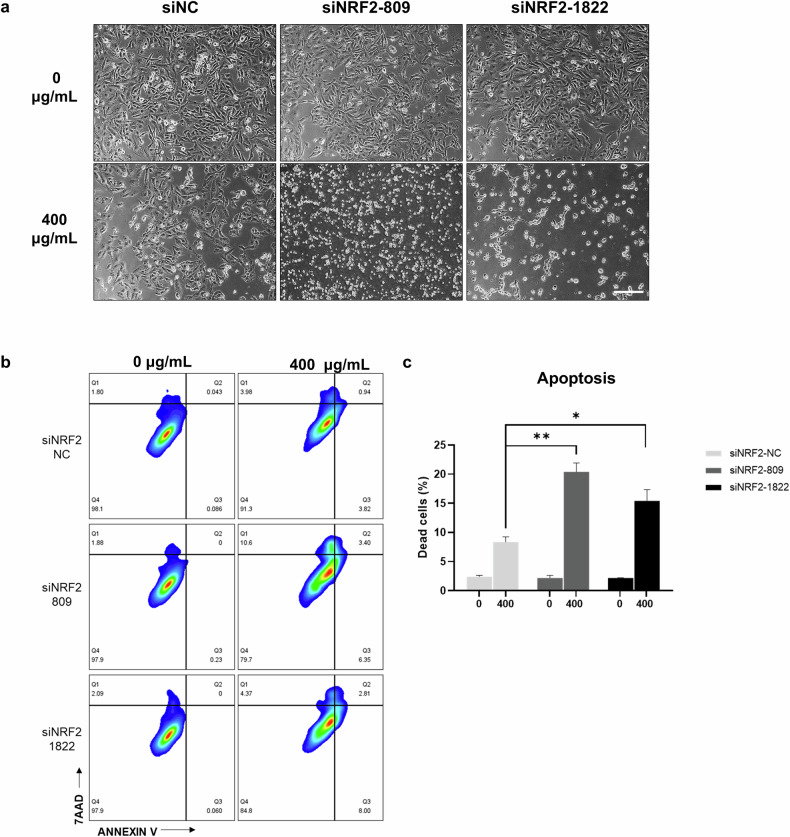

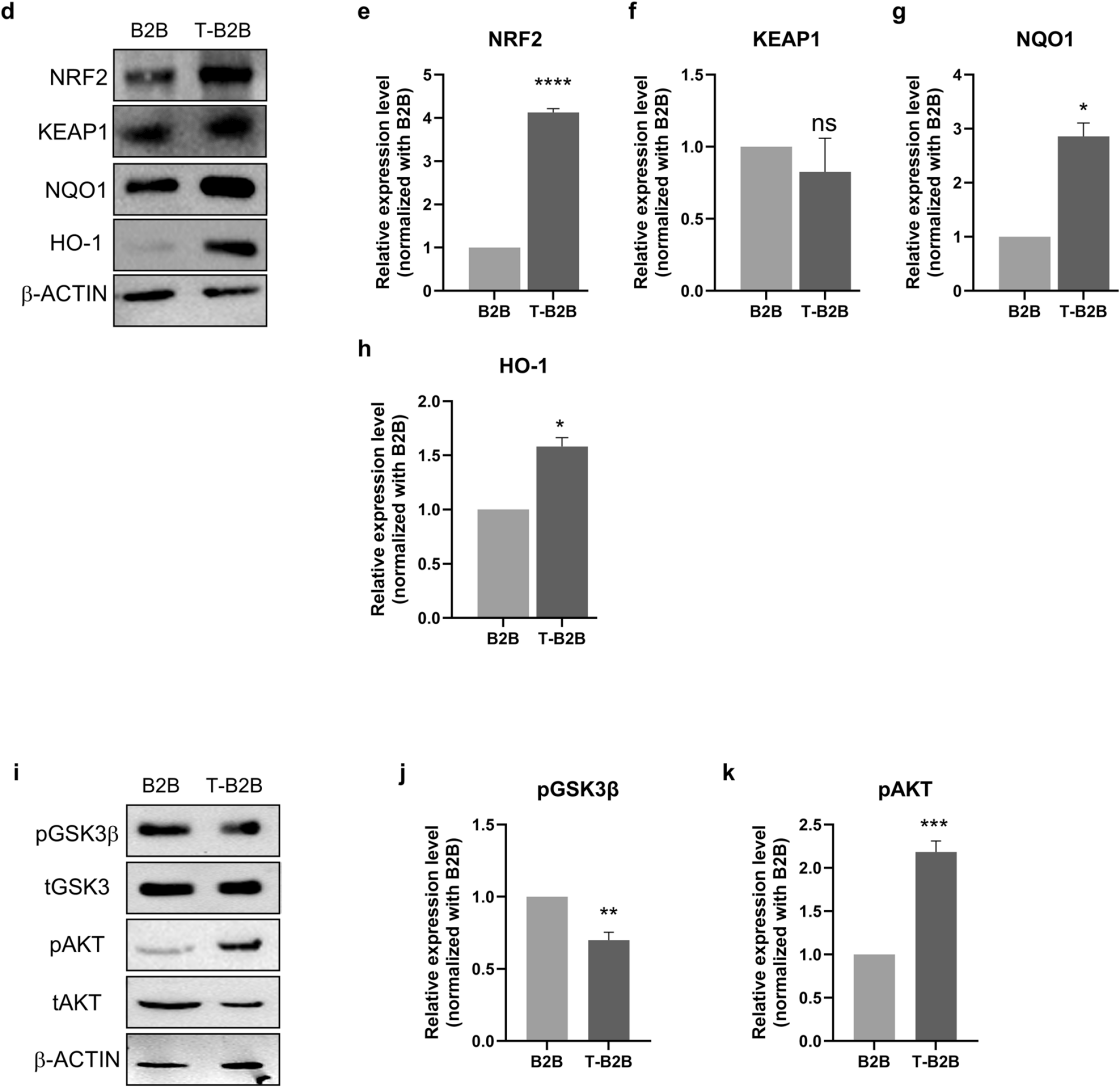
NRF2-dependent survival and AKT–GSK3β signaling under chronic WCSE exposure. **a**, **b** Representative bright-field images (**a**) and flow cytometry plots (**b**) showing apoptosis in T-B2B cells transfected with control siRNA (siNC), *NRF2*-targeting siRNAs (si*NRF2*-809 and si*NRF2*-1822) and exposed to 400 μg/ml WCSE for 24 h. **c** Quantification of apoptotic populations demonstrates that NRF2 knockdown significantly increased WCSE-induced cell death. **d** Representative western blot images of NRF2, KEAP1, NQO1, HO-1, and β-ACTIN in B2B and T-B2B cells. **e** Quantification of NRF2 protein levels. **f** Quantification of KEAP1 protein levels. **g** Quantification of NQO1 protein levels. **h** Quantification of HO-1 protein levels. **i** Representative western blot images of AKT–GSK3β signaling in B2B and T-B2B cells. **j** Quantification of inhibitory phosphorylated GSK3β (pGSK3β) levels. T-B2B cells exhibited reduced inhibitory pGSK3β levels relative to B2B. **k** Quantification of phosphorylated AKT (pAKT) levels. T-B2B cells exhibited higher pAKT levels relative to B2B, indicating sustained AKT activation and altered regulation of GSK3β. Data are presented as mean ± s.e.m. (*n* = 3). Statistical significance was analyzed by a *t*-test. Scale bar, 100 μm.

**Fig. 8:**
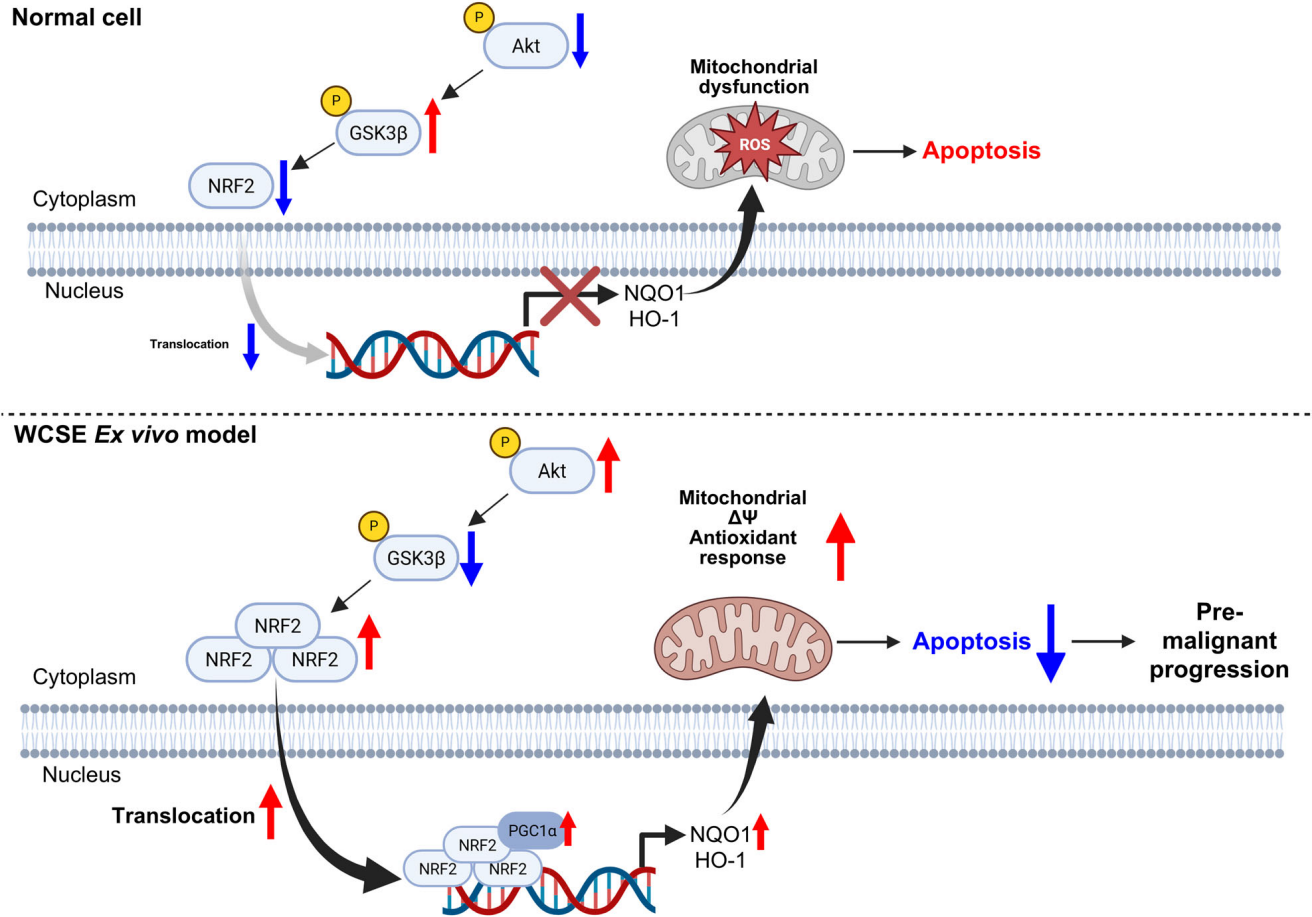
Proposed model of NRF2-dependent redox adaptation under chronic WCSE exposure. A schematic illustration summarizing the transition from acute oxidative injury to chronic adaptive remodeling. Top: in normal bronchial epithelial cells, acute WCSE exposure suppresses AKT activity and increases GSK3β activation, promoting NRF2 degradation and leading to elevated mitochondrial ROS and apoptosis. Bottom: by contrast, chronically exposed conditions increased AKT activation and inhibited pGSK3β, resulting in stabilization and nuclear accumulation of NRF2. Activated NRF2, together with PGC1α, enhanced the transcription of antioxidant and mitochondrial maintenance genes, preserving mitochondrial potential and suppressing apoptosis. These coordinated adaptations maintain redox balance and promote long-term survival under persistent CS-induced oxidative stress.

Our previous data indicated that chronically smoke-exposed cells acquire stress-resistant phenotypes characterized by the constitutive activation of NRF2. To confirm that NRF2 is essential for this acquired resistance, we performed *NRF2* expression knockdown using siRNA in T-B2B cells before acute WCSE exposure (Fig. 7a and Supplementary Fig. 6a–c). While cells transfected with a nontargeting control siRNA (siNC) were largely unaffected by 400 μg/ml WCSE, the knockdown of *NRF2* rendered the cells highly sensitive to WCSE, leading to apoptosis (Fig. 7b, c). This result confirms that NRF2 is essential for the survival of T-B2B cells under high oxidative stress. We examined the upstream regulatory pathway responsible for NRF2 activation. In T-B2B cells, acute WCSE exposure resulted in the constitutive upregulation of NRF2 and NQO1 independent of KEAP1 expression, suggesting that a KEAP1-independent mechanism contributes to the enhanced NRF2 stability in T-B2B cells (Fig. 7d–h). Recent studies have shown that glycogen synthase kinase-3β (GSK3β) negatively regulates the stability and nuclear localization of NRF2. By contrast, AKT-dependent phosphorylation of GSK3β suppresses its inhibitory effect, thereby enhancing NRF2 activation and antioxidant defense^20,21^. To investigate the upstream mechanism responsible for the improved NRF2 stability in T-B2B cells, we analyzed the AKT–GSK3β signaling axis. In parental B2B cells, the pro-survival kinase AKT was decreased in its active phosphorylated form (pAKT), whereas inhibitory phosphorylation of GSK3β was higher than in stress-resistant T-B2B cells. By contrast, stress-resistant T-B2B cells maintained high basal levels of active pAKT despite reduced levels of inhibitory pGSK3β (Fig. 7i–k), supporting KEAP1-independent stabilization and persistent antioxidant activation under chronic oxidative stress. Together, these findings define an adaptive signaling circuit in which long-term WCSE exposure reprograms the AKT–GSK3β–NRF2 axis to maintain redox homeostasis and promote survival (Fig. 8).

## Discussion

Continuous exposure to the complex oxidative environment of CS imposes persistent environmental stress that alters epithelial homeostasis through adaptive changes in redox, mitochondrial and transcriptional networks. In this study, we developed a chronic WCSE model that combines both particulate and gaseous components to better reflect the complexity of long-term exposure. Using this system in both immortalized bronchial epithelial cells and human lung organoids, we discovered that extended WCSE treatment induced consistent adaptive remodeling at both the cellular and tissue levels. Cells and organoids exposed to WCSE treatment chronically showed improved survival, reorganization of mitochondrial networks, and persistent activation of antioxidant defense pathways through NRF2 stabilization. Genomic analysis revealed oxidative stress-associated mutational patterns, suggesting that continuous exposure imprints both functional and genetic adaptations. Together, these findings suggest that chronic cigarette exposure activates a coordinated, multilayered stress adaptation program that maintains redox balance and metabolic stability, promoting sustained proliferation and long-term survival within the pulmonary system under oxidative pressure.

Prolonged WCSE exposure enhanced cell survival and shifted the cell cycle toward the S phase, indicating a proliferative adaptation rather than cumulative cytotoxicity. Although previous studies reported that CS stimulates proliferation through AKT signaling^8,21^, our results further reveal that chronic WCSE exposure suppresses apoptosis, suggesting an adaptive survival remodeling under persistent oxidative stress. The decreased apoptotic population observed in both 2D and organoid systems supports the idea that continuous oxidative pressure selects for stress-resistant subpopulations, consistent with early adaptive changes preceding premalignant adaptation in smoking-related airway lesions^19^.

Mitochondria act as both a major source and target of ROS produced by CS. In our WCSE model, mitochondria showed fragmented morphology but maintained membrane potential and had lower ROS levels, indicating controlled remodeling rather than dysfunction. These findings extend prior observations that chronic smoke exposure alters mitochondrial structure and respiration in airway epithelial and smooth muscle cells^22–24^. The observed fragmented yet functional mitochondria suggest a shift toward metabolic flexibility and antioxidant adaptation. Upregulation of PGC1α, a key regulator of mitochondrial biogenesis, further indicates a compensatory mitochondrial renewal process that helps sustain redox balance under persistent oxidative stress^9^.

WES identified oxidative stress-related mutational signatures, including SBS3, SBS5 and SBS18, which are enriched in smoking-related non-small cell lung cancers^20,25,26^. These patterns, resulting from ROS-induced DNA damage and imperfect repair, indicate that chronic oxidative stress leaves a lasting genomic imprint even before transformation occurs. Notably, while the 2D model primarily exhibited oxidative stress-associated signatures (SBS3, SBS5 and SBS18), the organoid system showed a shift toward the smoking-related SBS4 signature. This transition suggests that prolonged exposure within a three-dimensional epithelial context recapitulates mutational processes characteristic of smoking-related lung cancers, emphasizing that the tissue-level environment facilitates the emergence of canonical tobacco-associated genomic patterns. The detection of SBS4 (a hallmark of tobacco-induced mutagenesis) in the organoid model indicates that chronic WCSE exposure can elicit molecular alterations resembling those found in smoker-derived tumors, effectively bridging in vitro oxidative adaptation with in vivo mutational landscapes. Together, the presence of these signatures in nonmalignant, chronically exposed cells supports the idea that long-term cigarette exposure acts both as a genotoxic insult and as a selective pressure favoring cells capable of enduring genomic stress^20,26,27^.

A central mechanistic finding is that NRF2 activity persisted under chronic exposure without an increase in transcript levels. WCSE exposure elevated the NRF2 protein and its downstream targets, including *NQO1*, *HO-1*, *GCLC* and *PGC1α*, indicating post-translational stabilization. This is consistent with reports that GSK3β promotes NRF2 degradation, whereas AKT-mediated phosphorylation of GSK3β inhibits this process, thereby stabilizing NRF2 and enhancing its nuclear localization^11,20,26,28^. The observed elevation of pAKT and concomitant reduction of active GSK3β in chronically exposed cells indicate a KEAP1-independent mode of NRF2 regulation. Sustained NRF2 activation probably contributes to persistent antioxidant defense and cellular resilience during long-term oxidative challenge, consistent with observations that NRF2 signaling remains constitutively active in smoking-associated squamous carcinomas^29,30^. Moreover, the parallel induction of *PGC1α* and NRF2 target genes suggests a coordinated adaptation linking mitochondrial function to antioxidant defense. NRF2 and *PGC1α* have been reported to act cooperatively, maintaining redox homeostasis and supporting mitochondrial biogenesis^31,32^. In our model, the increase in PGC1α expression together with the maintained mitochondrial membrane potential suggests that chronic WCSE exposure promotes both mitochondrial and antioxidant adaptations. This NRF2–PGC1α axis may help maintain metabolic balance and redox homeostasis, allowing cells to tolerate continuous oxidative stress. Together, these results indicate that long-term CS exposure supports a redox–mitochondrial adaptive response that maintains cell survival under chronic oxidative conditions.

Our human lung organoid model reproduced these adaptive responses in a physiologically relevant three-dimensional system. Organoids chronically exposed to WCSE showed enlarged structures, a reduction in apoptotic cells and substantial nuclear accumulation of NRF2 while maintaining mitochondrial membrane potential. These features were consistent with those observed in chronically exposed bronchial epithelial cells, indicating that oxidative adaptation can also occur at the tissue level. The increased organoid size and decreased apoptosis suggest clonal expansion of stress-resistant cells, implying that epithelial adaptation to chronic oxidative stress may contribute to early remodeling processes within the lung.

Long-term in vitro exposure may promote the enrichment of stress-resistant subpopulations. In our study, experiments were performed using bulk populations, and the adaptive phenotypes were consistently reproduced across independent biological replicates and organoid systems, suggesting a population-level adaptive response under chronic WCSE conditions. Importantly, control cells, including BEAS-2B and human lung organoids, were passaged in parallel under the same culture conditions and duration as the WCSE-exposed cells, which allowed us to compare adaptive changes arising specifically from chronic smoke exposure rather than from prolonged culture alone. Nevertheless, prolonged exposure may contribute to clonal enrichment during culture. Future studies incorporating lineage-tracing or single-cell approaches may help clarify the dynamics of clonal adaptation under chronic CS exposure. The WCSE conditions used in this study reflect an experimental framework designed to model sustained oxidative stress in vitro. CS exposure in humans involves complex variables such as inhalation dynamics, metabolic processing and tissue clearance that are difficult to replicate in cell culture systems. Future studies incorporating more physiologically representative exposure platforms may further improve the translational relevance of chronic CS exposure models.

Taken together, our data support a model in which chronic cigarette exposure establishes an adaptive feedback loop. Persistent oxidative stress induces mitochondrial fragmentation and genomic adaptation, while activation of the AKT–GSK3β–NRF2 axis stabilizes antioxidant defenses. This interconnected network helps epithelial cells survive oxidative and DNA-damage challenges, supporting clonal persistence and setting the stage for premalignant adaptation. The chronic WCSE model, combined with human lung organoids, captures the essential features of this transition, bridging the gap between acute in vitro assays and long-term pathological remodeling. These findings provide a robust methodological platform for tobacco research as well as mechanistic insights into how prolonged smoke exposure promotes epithelial adaptation, revealing NRF2-dependent resilience as a crucial early marker in smoking-related lung cancer development.

## Supplementary information

Supplementary Information
