# Publisher Correction: A chronic whole cigarette smoke extract model reveals redox–mitochondrial adaptation in human lung epithelial and organoid models

**DOI:** 10.1038/s12276-026-01829-6

**Published:** 2026-08-07

**Authors:** Joo-Eun Lee, Dahye Lee, Jihyun Lee, Sung-Joon Han, Sung Hyun Kang, Ryeo-Eun Go, Jihyun Kwon, Younjhin Ahn, Mi Jung Lim, Mahn Jae Lee, Hee Min Yoo, Da Hyun Kang, Jeong Eun Lee, Dongil Park, Chaeuk Chung

**Affiliations:** 1 Division of Pulmonology and Critical Care Medicine, Department of Internal Medicine, College of Medicine, Chungnam National University, Daejeon, Republic of Korea; 2 Biomedical Convergence Research Institute, Chungnam National University Hospital, Daejeon, Republic of Korea; 3 Division of Thoracic and Cardiovascular Surgery, College of Medicine, Chungnam National University, Daejeon, Republic of Korea; 4 Division of Climate Change and Health Hazard, Department of Health Hazard Response, Korea Disease Control and Prevention Agency, Cheongju, Republic of Korea; 5 Genomics Department, Keyomics Co. Ltd., Yuseong-gu, Republic of Korea; 6 Biometrology Group, Korea Research Institute of Standards and Science (KRISS), Daejeon, Republic of Korea; 7 Department of Precision Measurement, University of Science and Technology (UST), Daejeon, Republic of Korea

**Keywords:** Mechanisms of disease, Experimental models of disease

Correction to: *Experimental & Molecular Medicine* https://doi.org/10.1038/s12276-026-01743-x, published online 8 June 2026

After online publication of this article, the authors noticed an error in the author information. Dongil Park was inadvertently omitted as a corresponding author. Dongil Park and Chaeuk Chung should have been designated as co-corresponding authors.

The correct corresponding author information should have read as below.

“In the author list, the correspondence symbol should appear after both Dongil Park and Chaeuk Chung. Correspondence and requests for materials should be addressed to Dongil Park (rahm3s@gmail.com) or Chaeuk Chung (universe7903@gmail.com).”

The authors apologize for any inconvenience caused.

The original article has been corrected.

