# Supplementary material for: A chronic whole cigarette smoke extract model reveals redox–mitochondrial adaptation in human lung epithelial and organoid models
Source: Exp Mol Med. 2026 Jun 8;58(6):1953–70. doi: 10.1038/s12276-026-01743-x (PMC13324337; doi:10.1038/s12276-026-01743-x)
Supplement: Supplementary file 1 — Supplementary Information [file 12276_2026_1743_MOESM1_ESM.pdf]

A chronic whole cigarette smoke extract model reveals redox-mitochondrial adaptation in human lung epithelial and organoid models

Joo-Eun Lee[1,2†], Dahye Lee[1†], Jihyun Lee[1], Sung-Joon Han[3], Sung Hyun Kang[4], Ryeo-Eun Go[4], Jihyun Kwon[4], Younjhin Ahn[4], Mi Jung Lim[5], Mahn Jae Lee[1,2], Hee Min Yoo[6,7], Da Hyun Kang[1], Jeong Eun Lee[1], Dongil Park[1*], and Chaeuk Chung[1*]

Supplementary Fig. 1

a.

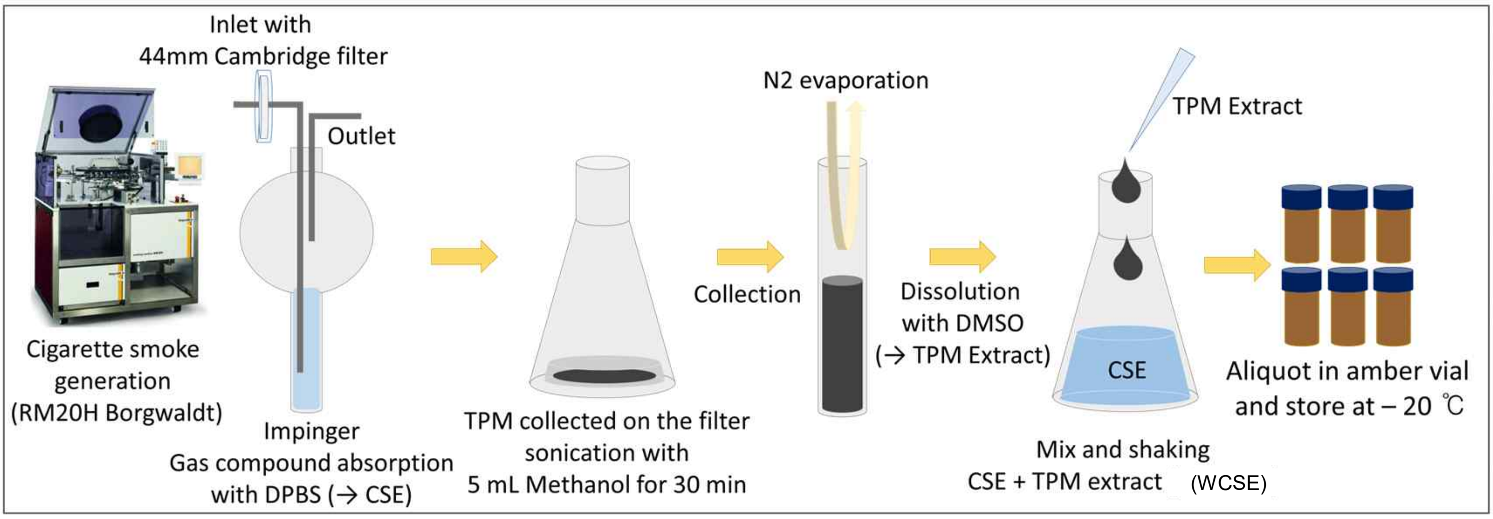

Supplementary Figure 1. Preparation of whole cigarette smoke extract (WCSE). (a) Schematic representation of WCSE generation combining both gaseous and particulate phases of mainstream cigarette smoke.

Supplementary Figure 2

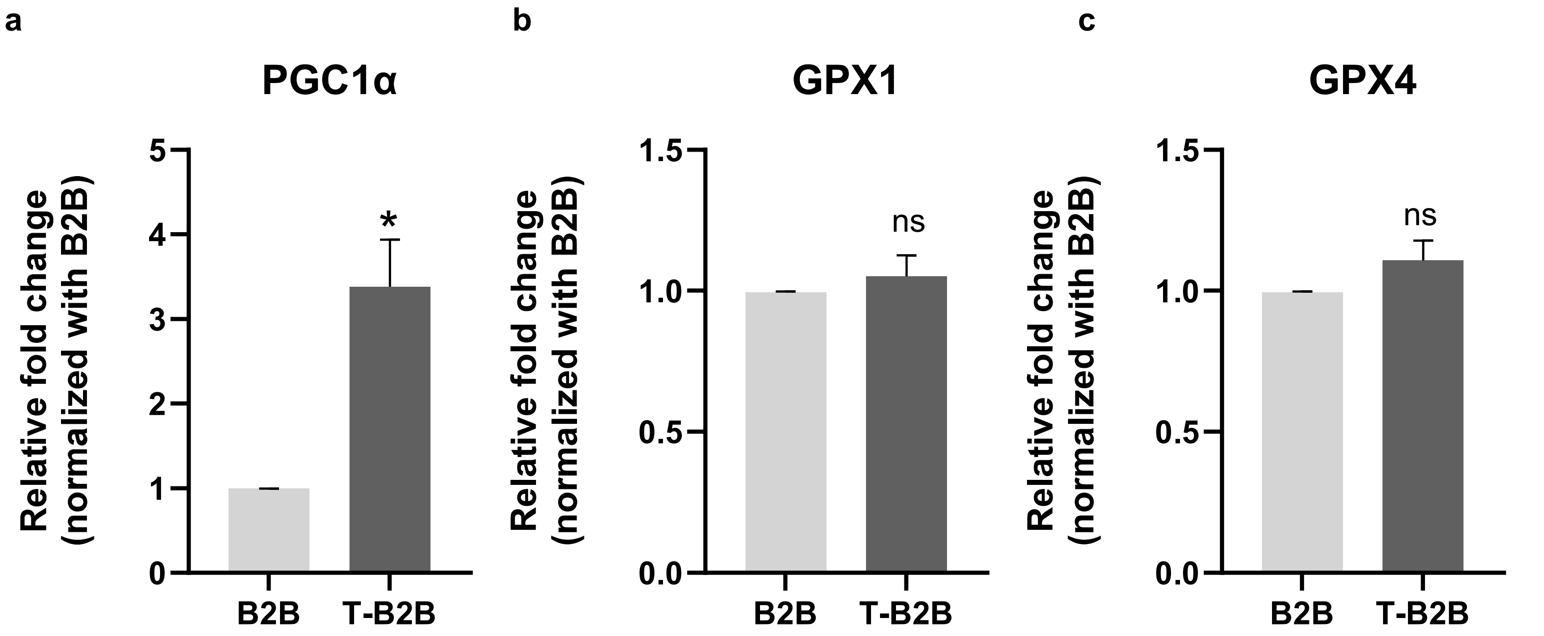

Supplementary Figure 2. Validation of antioxidant-related gene expression in chronic WCSE-exposed cells. (a-c) Quantitative qRT-PCR analysis of *PGC1α* (a), *GPX1* (b), and *GPX4* (c) mRNA levels in B2B and T-B2B cells. Data are presented as mean ± SEM (n=3). Statistical significance was analyzed by a *t-test*.

Supplementary Figure 3

a

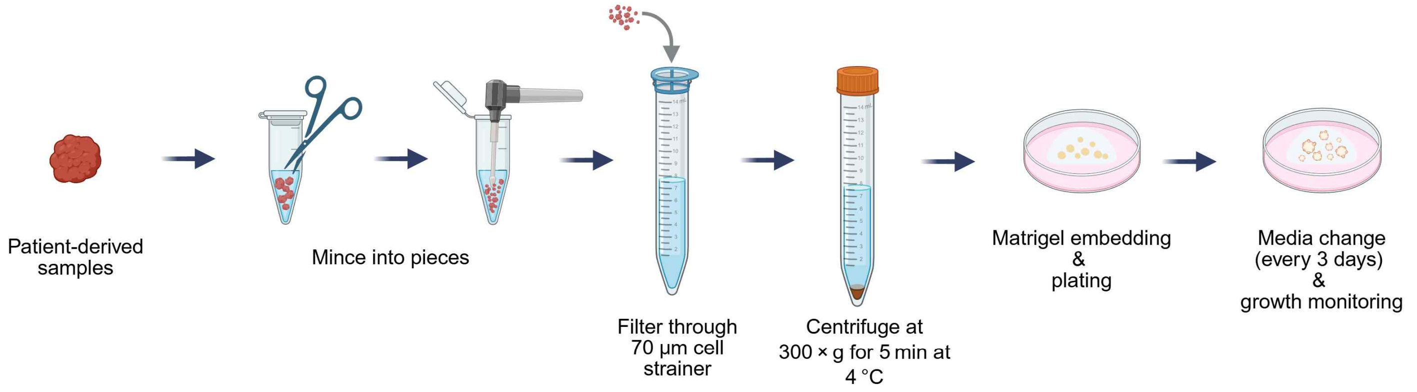

Supplementary Figure 3. Establishment of human lung organoid cultures from patient-derived lung tissues. (a) Schematic illustration of the workflow used to generate human lung organoids in this study. Lung tissues were minced, dissociated, filtered through a 70 μm strainer, centrifuged, and embedded in basement membrane extract (BME) for organoid culture. Organoids were maintained in a lung organoid medium with media changes every 3 days, and monitored for growth.

Supplementary Figure 4

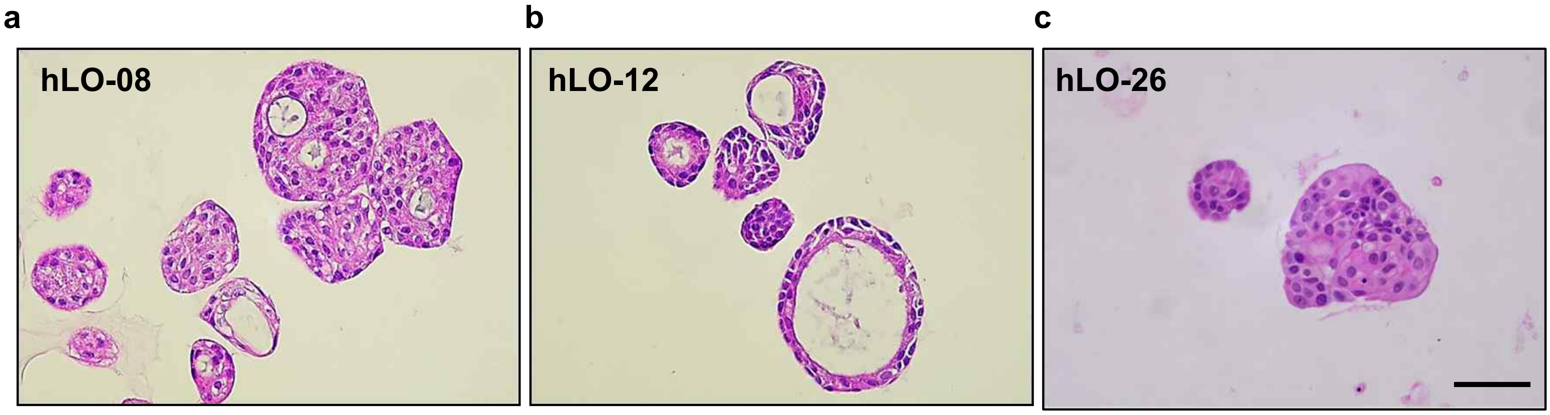

Supplementary Figure 4. Histological characterization of human lung organoids derived from independent donors. (a-c) Representative hematoxylin and eosin (H&E) staining images of human lung organoids established from three independent donors (hLO-08, hLO-12, and hLO-26) used in this study. Scale bar: 50 µm

Supplementary Figure 5

a

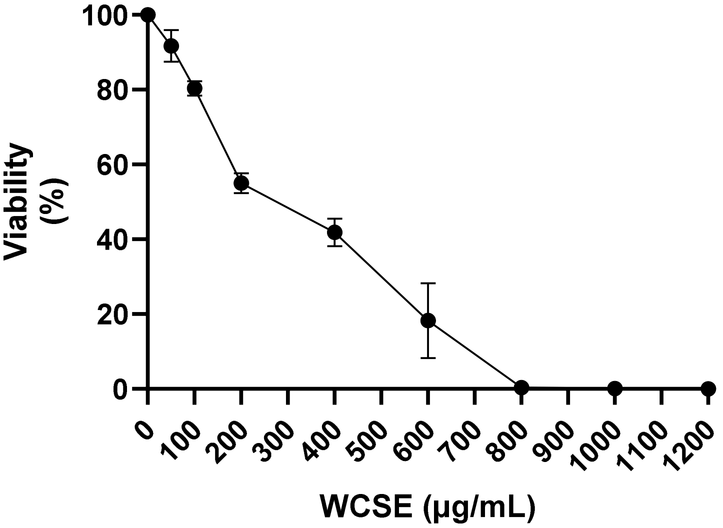

Supplementary Figure 5. Dose-response analysis of WCSE in human lung organoids. (a) Human organoids were exposed to increasing concentrations of whole cigarette smoke extract (WCSE) for the indicated period, and cell viability was measured using a 3D-Titer Glo assay. Data are presented as mean ± SEM (n=3 independent donor-derived organoid lines).

Supplementary Figure 6

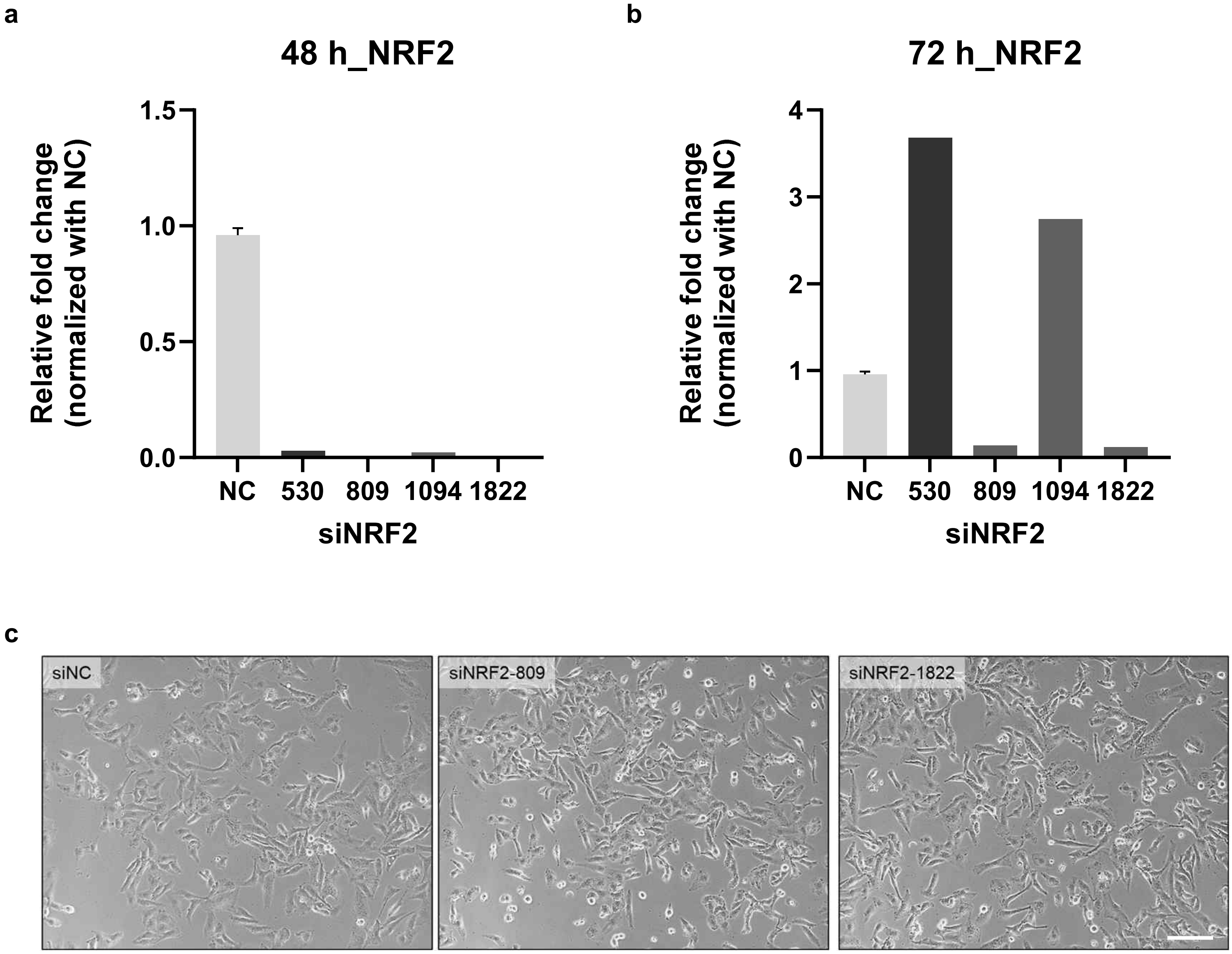

Supplementary Figure 6. Validation of NRF2 knockdown efficiency in T-B2B cells. (a, b) qRT-PCR analysis showing relative NRF2 mRNA levels 48 h (a) and 72 h (b) after transfection with control siRNA (siNC) or four different NRF2-targeting siRNAs (siNRF2-530, siNRF2-809, siNRF2-1094, and siNRF2-1822). Expression levels were normalized to the NC group. (c) Representative bright-field images showing the morphology of T-B2B cells 72h post-transfection with siNC, siNRF2-809, and siNRF2-1822.Scale bar: 100 μm.

Supplementary Figure 7

a

b

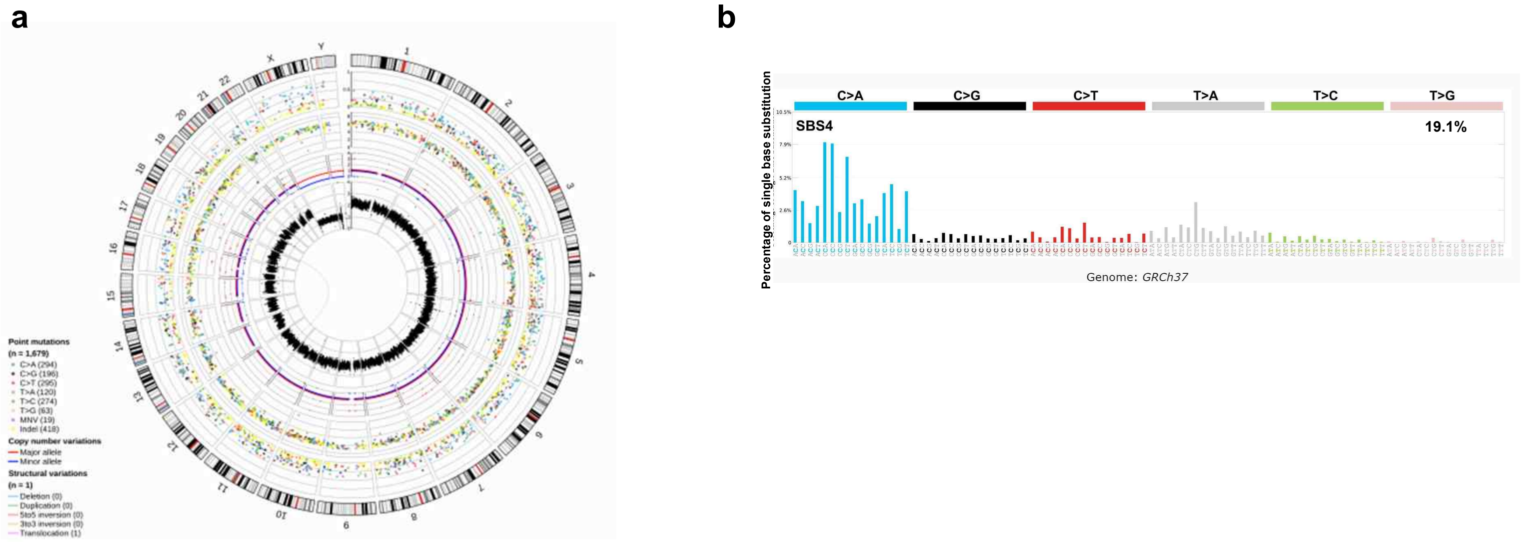

Supplementary Figure 7. Genomic characterization of long-term WCSE-exposed human lung organoids. (a) Circos plot indicating the distribution of somatic variants identified by whole-genome sequencing (WGS) in a representative chronically WCSE-exposed human lung organoid (T-hLO). Each track represents variant types and chromosomal locations. (b) Mutational signature analysis showing enrichment of the SBS4 signature, a hallmark of tobacco smoke-related mutagenesis, in T-hLOs.

Supplementary Tables

Supplementary Table 1.

Reagents	Source	Catalog No.	Final Conc.
AdDMEM/F12	Thermo Fisher Scientific (Gibco)	12634-010	
Pen/Step	Thermo Fisher Scientific (Gibco)	15070-063	1X
HEPES	Thermo Fisher Scientific (Gibco)	15630-080	10mM
Glutamax	Thermo Fisher Scientific (Gibco)	35050-061	1X
N-acetylcysteine	Sigma-Aldrich	A9165	1.25mM
Nicotinamide	Sigma-Aldrich	N0636	5mM
Primocin	InvivoGen	Ant-pm-1	50ug/ml
R-spondin1	PeproTech	120-38	500 ng/ml
hNoggin	PeproTech	120-10C	100ng/ml
hFGF7	PeproTech	100-19	25ng/ml
hFGF10	PeproTech	100-26	100ng/ml
B27 supplement	Thermo Fisher Scientific (Gibco)	17504-044	1X
SB202190	Sigma-Aldrich	S7067	500nM
A83-01	Tocris Bioscience	2939	500nM
Y27632	STEMCELL	72308	10uM

Supplementary Table 1. Composition of the human lung organoid culture medium. Components and working concentrations of the organoid medium used for the establishment and maintenance of human lung organoids in this study.

Supplementary Table 2.

Organoid ID	Histology	Sex	Age
hLO-08	Normal	M	65
hLO-12	Normal	M	71
hLO-26	Normal	M	63

Supplementary Table 2. Donor characteristics of human lung tissues used for organoid generation. Clinical information of donated human lung tissues to establish lung organoid cultures in this study.
